# Evolutionary Processes Involved in the Emergence and Expansion of an Atypical *O. sativa* Group in Madagascar

**DOI:** 10.1186/s12284-021-00479-8

**Published:** 2021-05-20

**Authors:** Nourollah Ahmadi, Alain Ramanantsoanirina, João D. Santos, Julien Frouin, Tendro Radanielina

**Affiliations:** 1 CIRAD, UMR AGAP, TA-A 108/03, Avenue Agropolis, F-34398 Montpellier Cedex 5, France; 2 AGAP, Univ Montpellier, CIRAD, INRA, Montpellier SupAgro, Montpellier, France; 3 FOFIFA, Ampandrianomby, B.P. 1690, 101 Antananarivo, Madagascar; 4 Université de Antananarivo, département de biologie et écologie végétale, Antananarivo, Madagascar

**Keywords:** Rice, *Oryza sativa*, Madagascar, Diversity, Evolutionary process

## Abstract

Understanding crops genetic diversity and the evolutionary processes that accompanied their worldwide spread is useful for designing effective breeding strategies. Madagascar Island was one of the last major Old World areas where human settlement brought the introduction of *Oryza sativa*. Early studies in the island had reported the presence of a rice group specific to Madagascar. Using 24 K SNP, we compared diversity patterns at the whole genome and at haplotype (30 SNP-long segments along the genome) levels, between 620 Malagasy and 1929 Asian rice accessions. The haplotype level analysis aimed at identifying local genotypic variations, relative to the whole genome level, using a group assignment method that relies on kernel density estimation in a Principal Component Analysis feature space. Migration bottleneck had resulted in 10–25% reduction of diversity among the Malagasy representatives of *indica* and *japonica* populations. Compared to their Asian counterpart, they showed slightly lower *indica* and *japonica* introgressions, suggesting the two populations had undergone less recombination when migration to the island occurred. The origins of the Malagasy *indica* and *japonica* groups were delineated to *indica* subpopulation from the Indian subcontinent and to tropical *japonica* from the Malay Archipelago, respectively. The Malagasy-specific group (*Gm*) had a rather high gene diversity and an original haplotype pattern: much lower share of *indica* haplotypes, and much higher share of *Aus* and *japonica* haplotypes than *indica*. Its emergence and expansion are most probably due to inter-group recombination facilitated by sympatry between *indica*-Aus admixes and “Bulu” type landraces of *japonica* in the central high plateaux of Madagascar, and to human selection for adaptation to the lowland rice cultivation. Pattern of rice genetic diversity was also tightly associated with the history of human settlement in the island. Emergence of the *Gm* group is associated with the latest arrivals of Austronesians, who founded the Merina kingdom in the high plateaux and developed lowland rice cultivation. As an intermediary form between *Aus*, *indica* and *japonica*, the three pillars of *O. sativa* domestication, *Gm* represents a very valuable genetic resource in breeding for adaptation to cold tolerance in tropical highlands. We proposed the name *Rojo* for this new rice group.

**Supplementary Information:**

The online version contains supplementary material available at 10.1186/s12284-021-00479-8.

## Introduction

Rice, *Oryza sativa* L., displays very large morphological and physiological variations contributing to its cultivation as a food crop in a very large range of environments (Maclean et al., 2002). This phenotypic diversity is associated with differentiation into two subspecies, *indica* and *japonica* (Oka, 1983), and at least two minor ecotypes, *Aus* and *Basmati*, distinguishable by isozyme (Glaszmann, 1987) and DNA markers (Garris et al., 2005). Recently, analysis of population structure of *O. sativa*, from the 3000 Rice Genomes Project (3 K-RG, 2014), identified nine subpopulations, of which four belonged to the *indica* subspecies, three to the *japonica* subspecies, and the remaining two encompassed the *Aus* and *Basmati* ecotypes. The differentiation of the subpopulations was associated with some eco-geographical specialisation. The *indica* subpopulations named XI-1A, XI-2 and XI-3 mainly originated from East Asia, South Asia and Southeast Asia, respectively. The subpopulation XI-1B assembled modern *indica* varieties of diverse origins. The *japonica* subpopulations named GJ-trop, GJ-sbtrop and GJ-temp mainly originated from tropical Southeast Asia, subtropical Southeast Asia, and temperate East Asia, respectively. The subpopulations encompassing *Aus* and *Basmati* ecotypes were named cAus and cBas, respectively (Wang et al., 2018).

While the domestication of *O. sativa* goes back to an estimated 10,000 years (Higham and Lu 1998; Sweeney and McCouch, 2007), the seniority of *indica*-*japonica* differentiation within its wild ancestor *O. rufipogon* has been estimated at more than 100,000 years (Wang et al., 1992; Ma and Bennetzen, 2004). Based on this early differentiation, it was often concluded that *O. sativa* had undergone two independent domestications from the divergent pools of *O. rufipogon* (Second, 1982; Cheng et al., 2003; Vitte et al., 2004). However, Molina et al. (2011), analysing the polymorphism of 630 gene fragments, using a demographic modelling approach, concluded to a single origin of Asian domesticated rice. Likewise, Huang et al. (2012), analysing the domestication sweeps and genome-wide patterns in genome sequences of 446 accessions of *O. rufipogon* and 1083 accessions of *O. sativa*, concluded that *japonica* rice was first domesticated from a specific population of *O. rufipogon* in southern China. *Indica* rice was subsequently developed from crosses between *japonica* rice and local wild rice as the initial cultivars spread into South East and South Asia. On the other hand, considering the domestication not as an event but as an evolutionary process promoted by interactions between plant and man, Oka (1988) suggested multiple and diffuse domestications of *O. sativa*, in a large area stretching from the Himalayan footsteps of India to China. Glaszmann (1988), reporting marked isozymic differentiation among minor ecotypes, such as *Aus* and *Aromatic*, in the Indian subcontinent, reinforced the assumption of diffuse domestications and the major contribution of local wild forms. The fact that these ecotypes contained unique alleles not found in *indica* and *japonica* was later confirmed by Jain et al. (2004) and Garris et al. (2005) using SSR markers. Likewise, a recent reinterpretation of Huang et al. (2012) data concluded on three sources of domestication, distributed across the *Indica*, *Japonica* and *Aus*, and the Basmati-like cluster to be a hybrid between *Aus* and *Japonica* (Civan et al., 2015). However, the presence of some weedy rice among the plant material somewhat compromises this interpretation. The hypothesis of multiple independent domestication events was further reinforced by the analysis of patterns of introgressions in nine important domestication genes in the 3 K-RG accessions (Wang et al., 2018).

Understanding the evolutionary processes that accompanied rice domestication and spread has been complicated in Asia (i) by large-scale migration of cultivated rice accompanying human movements across the continent in areas where wild ancestor populations were already present, (ii) by recombination between different *O. sativa* subpopulations (Kovach et al.*,* 2009, Santos et al., 2019) despite some level of reproductive barrier, and (iii) more recently, by the trading of cultivated rice varieties. Analysis of rice diversity pattern in areas where *O. rufipogon* is absent may provide insights into *O. sativa* expansion processes. The Madagascar Island represents a good case of such an expansion area. According to human genetic data, the island’s human settlement of Indonesian origin goes back most probably between 1400 and 1200 years ago (Tofanelli et al., 2009; Cox et al., 2013). Rice is the country’s staple food and is grown wherever possible with very ingenious developments in inland valleys and terraces on hillsides. Among the wild relatives of *O. sativa*, so far only the presence of *O. longistaminata* and *O. punctata* has been reported (FAO, 1996); and the African cultivated rice, *O. glaberrima*, is absent from the island.

Studying the variability of morpho-physiological traits in the national collection of rice varieties established in the 1940s and conserved ex situ by the national agronomic research institute (FoFiFa), Ahmadi et al. (1988) identified, in addition to representatives of *indica* and *japonica* subspecies, an atypical group specific to Madagascar and preferentially present in the central high plateaux of the country. Compared to the *indica* landraces, the atypical landraces were taller, had a larger stem diameter, longer and larger leaves, longer panicles, shorter but wider grains and a shorter growth cycle. The finding of an atypical group was confirmed later using isozymic data (Ahmadi et al.*,* 1991). More recently, Mather et al. (2010), using sequence data from 53 gene fragments, reported a clear differentiation of Madagascar *indica*-type landraces from Asian *indica*, as well as evidence of *indica/japonica* recombinations. Likewise, Radanielina et al. (2012), analysing the in situ eco-geographical distribution of *O. sativa* in the highlands of Madagascar, using SSR markers, confirmed the presence of the atypical group and reported a high level of within-variety diversity. Understanding the evolutionary processes (e.g. founder events, hybridisation and selection) that accompanied the introduction and spread of rice in Madagascar will also affect the conservation and utilisation strategies for the valuable genetic resources it represents.

The availability of the 3 K-RG project data and the feasibility of producing dense genotypic data for a large set of Malagasy rice accessions provided us with the opportunity to analyse the original diversity of the Malagasy rice gene pools on a fine scale. Here, using a 24 K SNP dataset, we describe the pattern of rice diversity in Madagascar relative to the reference pattern observed in Asia. The observed pattern confirmed the presence in Madagascar of a group not identifiable as such in Asia. The evolutionary processes most probably involved in the emergence and expansion of this original ecotype are (i) multiple hybridisations and recombinations between *indica* and cAus subpopulations in the context of their sympatry in south Asia; (ii) multiple hybridisations and recombinations between the *indica*-cAus intermediary form that reached Madagascar with lowland-grown tropical *japonica* subpopulation in the context of their sympatry in the highlands of the country; (iii) favourable selection pressure for the survival of the recombinant forms in the cold climate of the island’s high plateaux.

## Materials and Methods

### Plant Material

The study included a Malagasy diversity panel and an Asian reference panel. The Malagasy panel was composed of 620 Malagasy rice landraces belonging to the Malagasy national collection of rice varieties. Among those accessions, 410 (111 collected before 1980, and 299 collected in 2012) represented the countrywide rice diversity. The remaining 210 accessions were collected in 2007, in the high-plateau (Vakinankaratra) region of the country (Supplementary Table 1). Each accession of the Malagasy panel was georeferenced. The Asian reference panel was composed of 1929 Asian rice landraces. They were extracted from the list of over 3000 accessions that were re-sequenced in the framework of the 3000 rice genomes project (3 K RGP, 2014). The extraction was based on two selection criteria applied successively. The first criterion was the geographic origin. Only accession of Asian origin was retained. The second criterion was the status, landrace versus improved, of the accessions. Only accessions with a high likelihood of being a landrace (based on the structure of the name) were retained. According to the Wang et al. (2018) classification of the 3 K RGP accessions, among the 1929 accessions of our Asian panel, 1139 belong to *indica*, 495 to *japonica*, 169 to cAus, and 62 to cBas subpopulations. The remaining 64 were considered as Admix (Supplementary Table 2). Hereafter we will refer to the Malagasy panel as M-panel, to the Asian panel as A-panel, to Asian ensembles as subpopulations and to Malagasy ensembles as groups.

### Genotypic Data

The M-panel was genotyped using the genotyping by sequencing (GBS) methodology. DNA libraries were prepared at the Regional Genotyping Technology Platform (http://www.gptr-lr-genotypage.com) hosted by Cirad, Montpellier, France. For each accession, genomic DNA was extracted from the leaf tissues of a single plant, using the MATAB method. Each DNA sample was diluted to 100 ng/μl and digested separately with the restriction enzyme *ApekI*. DNA libraries were then single-end sequenced in a single-flow cell channel (i.e. 96-plex sequencing) using an Illumina HiSeq™2000 (Illumina, Inc.) at the Regional Genotyping Platform (http://get.genotoul.fr/) hosted by INRA, Toulouse, France. The fastq sequences were aligned to the rice reference genome, Os-Nipponbare-Reference-IRGSP-1.0 with Bowtie2 (default parameters). Non-aligning sequences and sequences with multiple positions were discarded. Single nucleotide polymorphism (SNP) calling was performed using the Tassel GBS pipeline v5.2.29. (Bradbury et al., 2007). The initial filters applied were the quality score (> 20), the count of minor alleles (> 1), and the bi-allelic status of SNPs. In the second step, loci with minor allele frequency (MAF) below 2.5%, with heterozygosity rate > 5% and with more than 20% missing data were discarded. The missing data were imputed using Beagle v4.0. The process yielded 34,614 SNP, which represents an average marker density of one SNP every 13.2 kb.

This working dataset can be downloaded in HapMap format from http://tropgenedb.cirad.fr/tropgene/JSP/interface.jsp?module=RICE study Genotypes, study type M-panel_GBS_data.

Genotypic data for the A-panel was extracted, during December 2018, from the SNP-Seek database (http://snp-seek.irri.org/; Mansueto et al. 2017), which contained over 29 million bi-allelic markers from 3 K-RG project. The selection criteria were: (i) matching with one of the 34,614 SNP obtained through GBS for the M-panel, (ii) rate of missing data < 20%, (iii) heterozygoty < 5% and (iv) MAF > 1%. The selection process yielded 23,981 SNPs, which represents an average marker density of one SNP every 18.8 kb. The missing data were imputed using Beagle v4.0. The heat map of distribution of the two genotypic datasets along the chromosomes is presented in Supplementary figure 1.

### Analysis of the Genotypic Data

Analyses of genotypic data aiming at characterisation of the genetic diversity of the M-panel, per se, were performed with the 34,614 SNPs data set. Analyses of genotypic data aiming at characterisation of the genetic diversity of the M-panel, relative to the genetic diversity of the A-panel were performed with the 23,981 common SNPs, hereafter referred to as 24 k SNPs.

#### Whole-Genome Level Analyses

The structure of the two panels were first analysed separately, then jointly. In each case, estimates of ancestry coefficients were obtained, using the inference algorithm “sparse nonnegative matrix factorization” (sNMF) (Frichot et al., 2014). The sNMF algorithm produces estimates of ancestry proportions similar to STRUCTURE (Pritchard et al., 2000) or ADMIXTURE (Alexander and Lange, 2011) with much shorter runtimes. The algorithm was implemented under the R package LEA (Frichot and Francois, 2015).

The distances between individual accessions were investigated using a simple-matching dissimilarity index, with DARwin software (Perrier and Jacquemoud-Collet, 2006). An unweighted neighbour-joining tree was constructed based on this dissimilarity matrix.

Differentiation between populations was estimated using pairwise *F*_*ST*_ which estimates genetic differentiation based on allele frequency (Wright, 1931). The *F*_*ST*_ statistics were calculated over the 23,981 SNPs common to M-panel and A-panel, using Arlequin 3.5.2.2 software (Schneider et al., 2000). The same software was used to estimate gene diversity within each population. Gene diversity was defined as the probability that two randomly chosen haplotypes are different in the sample and was estimated as $\overset{`}{\hat{H}=\frac{n}{n-1}\ \left(1-{\sum}_{i=1}^k{P}_i^2\right)}$, where *n* is the number of gene copies in the sample, k is the number of haplotypes, and *P*_*i*_ is the sample frequency of the i-th haplotype (Nei, 1987).

The speed of decay of linkage disequilibrium (LD) in the two panels was estimated by computing r^2^ between pairs of markers on a chromosome basis, using the “full matrix” option of Tassel 5.2.63 software (Bradbury et al., 2007), and then by averaging the results by classes of distance using XLSTAT.

#### Characterisation of Local Haplotypes

This characterisation aimed at identifying local, haplotype level, genotypic variations among the accessions, relative to the global, whole genome level, classification resulting from the above-described whole genome analyses. We implemented the characterisation and assignment method developed by Santos et al. (2019). It relies on kernel density estimation (KDE) in a principal component analysis (PCA) feature space. KDE is a non-parametric method that estimates the density of probability of random variables. It produces an approximation of the distribution of data in the form of combination of kernels (McKinney, 2012). Briefly, first a PCA is performed using the genotypic data of all accessions under study (in our case M-panel and A-panel) at a given number of adjacent SNP loci of a given chromosome. Then, for each accession, the first five coordinates of the PCA are submitted to the KDE algorithm to compute the likelihood of membership of the accession to each of the reference populations predefined at the whole genome level. Finally, the confrontation of the membership likelihoods computed by the KDE algorithm to a predefined threshold leads to assignment of the local haplotype to one of the reference populations or to one of the haplotypes intermediate between the reference populations, or to an unknown, outlier, haplotype. The process is repeated systematically along each chromosome. In our case, the number of reference populations retained was three, i.e., the accessions belonged to either *indica*, or *japonica* or cAus populations, or were classified as admixed. In other words, accessions identified as cBas in Wang et al. (2018) classification were considered as admix. This choice was in line with the three-pillar view of rice domestication (Civan et al., 2015) and offered the advantage of limiting the number of intermediary classes of haplotype to four. Thus, the final number of classes of haplotypes was equal to eight: *ind*, *jap*, cAus, *ind*-*jap*, *ind*-cAus, *jap*-cAus, indi-*jap*-cAus, and outlier. After several tests, the size of the beans retained was of 30 consecutive SNP loci. The overlap between two adjacent beans was of 15 SNPs loci. The local classification was implemented with the function *KernelDensity* of the python package *sklearn.neighbors* (Pedregosa et al. 2011) that was used with a Gaussian Kernel. The *p*-values ratio threshold for classification of the local haplotypes into the three reference classes and the four intermediate classes was set to 3. All local haplotypes, with *p*-values of assignment to each of the three reference classes below 0.0005 were classified as outliers.

Results of the local assignments were summarised in ideograms. For each accession and each chromosome, in order of increasing first SNP, windows of the same classification were merged into a single block. A new block was created with each change in class.

## Results

### Population Structure

The structure analysis performed on the 1929 accessions of the A-panel indicated K = 3 as the most likely number of populations, i.e., the lowest rate (10%) of accessions with ancestry coefficients below the threshold of 0.80. The three populations corresponded to the *indica*, *japonica* and cAus. Assignment of individual accessions to one of the three populations was almost completely in line with the Wang et al. (2018) classification of the 3 K RGP accessions, based on ~ 4.8 million SNPs. The rate of identical classification was of 96% for the *indica* population (1090 out of 1139), 100% for the *japonica* population (495 out of 495), and 92% for cAus (156 out of 169). The 187 admix accessions, with estimated ancestry coefficients below 0.80, included 90 accessions classified as Admix, XI-adm or GJ-adm by Wang et al. (2018), and 16, 62 and 19 accessions assigned by the same authors to cAus, cBas and *indica* populations, respectively. The 62 cBas accessions derived a large share (0.68%) of their ancestry coefficient from the *japonica* population (Supplementary figure 2). The hypotheses of K = 4 and K = 5 subdivided the *indica* population into its components XI-1, XI-3 and XI-2, and at K = 6, the cBas accessions formed a separate population. However, under these hypotheses, the rate of accessions with ancestry coefficients below 0.80 increased drastically, reaching 39%, 46% and 43%, respectively. Thus, one can consider that our 24 K SNP genotypic dataset provided a reasonably accurate description of the global structure of the A-panel.

**Fig. 1:**
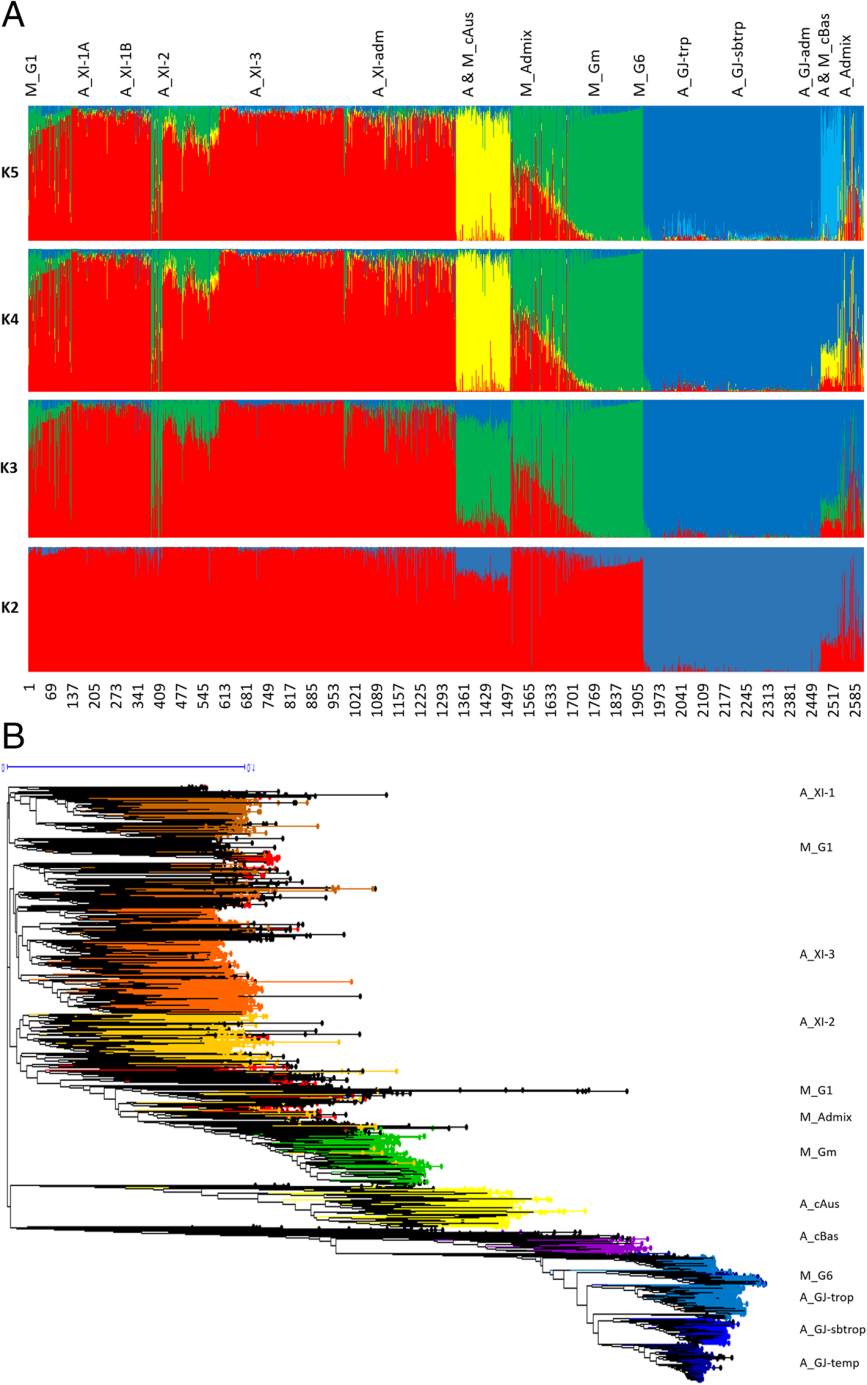
Population structure in 1929 Asian and 620 Malagasy rice accessions estimated from 23,981 genome wide SNPs. **a**: output of sNMF for K = 2 to K = 5. From top to bottom, the Asian accessions (prefix A) are organised first by their classification in Wang et al. 2018 groups and subgroups, then by alphabetic order of country (not shown). Malagasy accessions (prefix M) are organised first by groups identified by sNMF with K = 3, then by increasing values of ancestry coefficient. **b** Unweighted neighbour-joining tree of simple matching distances. Tree branches are coloured according to membership in sNMF groups at K = 3 and ancestry coefficient 0.8, for Malagasy accessions, according to Wang et al. (2018) subpopulations of Asian accessions

When a similar clustering analysis was performed on the A-panel plus M-panel of 620 accessions, the hypothesis of K = 2 separated a population including the *indica* accessions from another including the *japonica* accession (Fig. 1a). At K = 3, the population including the *indica* accessions split into 2, and at K = 4 it split into 3 subpopulations. At K = 5, the population including the *japonica* accessions split into 2 subpopulations. K values above five did not improve population discrimination and did not reduce the number of Admix. Among the populations identified at K = 5, four corresponded to *indica*, *japonica*, cAus and cBas from the corresponding A-panel, plus 21%, 7%, 0.2% and 0.3% of accessions from the M-panel, respectively. The fifth population, exclusively composed of Malagasy accessions (38% of the accessions of the M-panel), corresponded to the group specific to Madagascar first described by Ahmadi et al. (1988). Thirteen percent of accessions of A-panel and 33% of accessions of M-panel with less than 80% of their estimated ancestry deriving from one of the identified populations were considered as admixed.

**Fig. 2:**
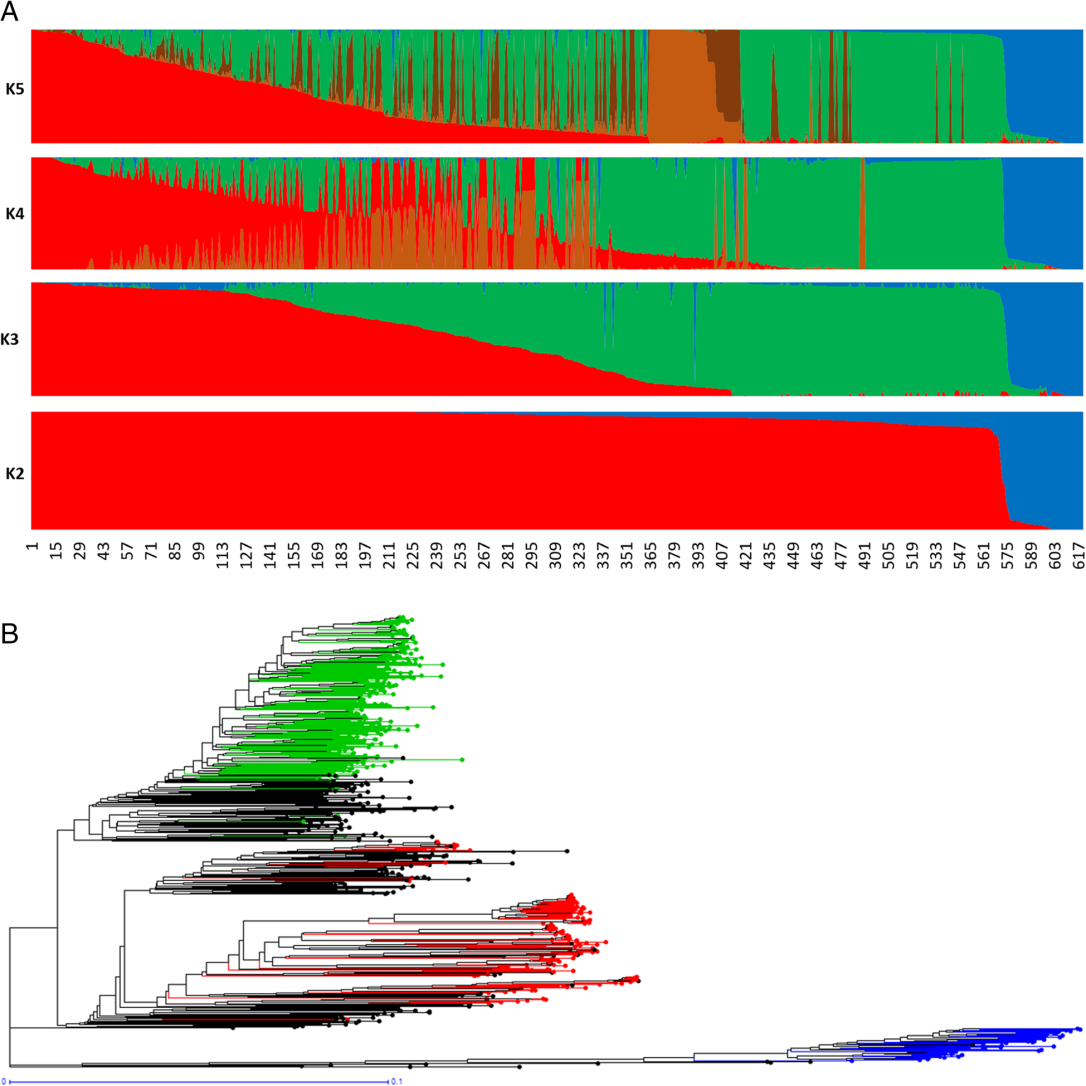
Population structure in 620 Malagasy rice accessions estimated from 32,614 SNPs. **a**: Output of sNMF for K = 2 to K = 5; **b**: Unweighted neighbour-joining tree of simple matching distances. Tree branches are coloured according to membership of sNMF groups at K = 3 and ancestry coefficient threshold of 0.8

Lastly, when structure analysis was performed on the 620 accessions of the M-panel alone, the most likely number of populations was K = 3, among which two corresponded to *indica* and *japonica* and the third to the group specific to Madagascar (Fig. 2a). The M-panel representatives of *indica*, hereafter called *G1* group, accounted for 25% of the accessions, the *japonica* accessions, hereafter called *G6* group, for 7%, and the group specific to Madagascar hereafter called *Gm* for 36%. The remaining 32% accessions were mainly admixtures of the *G1* and *Gm* groups, except two Malagasy aromatic varieties with more than 70% of their estimated ancestry deriving from *G6*, and one cAus accessions. The *G1* group consisted of lowland rice varieties cultivated in low elevation areas (altitudes < 800 m). The *G6* was composed of upland rice varieties cultivated in the low elevation forest areas of the east coast (altitude < 750 m) on the one hand, and a few lowland rice varieties cultivated in high elevation areas (altitude > 1250 m) on the other hand. Lastly, the *Gm* assembled lowland rice varieties mainly cultivated at altitudes between 800 m and 1600 m (Supplementary figure 3).

The distance-based neighbour-joining tree supported the results of the structure analyses for the joint analysis of the A-panel and M-panel (Fig. 1b) as well as for the analysis of the M-panel alone (Fig. 2b). It also allowed to separate rather clearly the component subpopulations of the *indica* and *japonica*, as subdivided by Wang et al. (2018). The *indica* XI-1A subpopulation mainly originated from China, the XI-1B subpopulation of diverse origins, the XI-2 subpopulation mainly originated from the Indian subcontinent, and the XI-3 subpopulation mainly originated from South-East Asia. The GJ-temp component mainly originated from Japan, China and Korea, the GJ-trop component, mainly originated from Indonesia, Philippines, Malaysia and Sri Lanka, and the GJ-sbtrop component mainly originated from Bhutan, India, Laos, Myanmar and Thailand (Fig. 1b). The Malagasy *G1* accessions split into two ensembles, one clustering with the Asian XI-1B subpopulation of very diverse origins, and the other, together with the Malagasy admix accessions, with the Asian XI-2 subpopulation of South-Asian origin. The Malagasy *G6* accessions formed a distinct cluster among the Asian GJ-trop subpopulation, mainly of Indonesian origin. The Malagasy *Gm* group formed a distinct cluster at the vicinity of the Asian XI-2 subpopulation.

### Genetic Characteristics of the Asian Subpopulations and Malagasy Groups

In the A-panel, average gene diversity over the 24 k common SNPs ranged from 0.030 ± 0.0142 for GJ-temp subpopulation to 0.177 ± 0.084 for XI-2 (Table 1). Average gene diversity in Malagasy *G1* (0.138 ± 0.089) and *G6* (0.100 ± 0.048) was smaller compared to their Asian counterpart *indica* (0.183 ± 0.086) and *japonica* (0.111 ± 0.037). The *Gm* group had a medium level gene diversity, 0.094 ± 0.044.

**Table 1:** Gene diversity, linkage disequilibrium and genetic differentiation between populations estimated by *F*_*ST*_ statistic

					Asian panel										Malagasy panel		
Population	N	Gene diversity	Distance^a^ (kb) for r^2^ ≤ 0.2	Genetic differentiation between subpopulations estimated by *F*_*ST*_ statistic													
				*indica*	XI-1A	XI-1B	XI-2	XI-3	cAus	*japonica*	GJ- trop	GJ-sbtrop	GJ-temp	cBas	*G1*	*Gm*	*G6*
*Indica* (XI)	1139	0.19 ± 0.09	75–100														
- XI-1A	178	0.13 ± 0.06	250–300		0.00												
- XI-1B	49	0.15 ± 0.07	250–300		0.25	0.00											
- XI-2	179	0.18 ± 0.08	125–150		0.23	0.17	0.00										
- XI-3	392	0.16 ± 0.06	125–150		0.25	0.18	0.15	0.00									
cAus	169	0.16 ± 0.08	200–250	0.42	0.53	0.49	0.42	0.49	0.00	0.73							
*japonica* (GJ)	495	0.11 ± 0.04	350–400	0.66													
- GJ-trop	210	0.09 ± 0.04	500–600		0.74	0.74	0.68	0.68	0.70		0.00						
- GJ-sbtrop	105	0.05 ± 0.02	700–800		0.76	0.78	0.69	0.69	0.71		0.25	0.00					
- GJ-temp	150	0.03 ± 0.01	700–800		0.79	0.83	0.72	0.71	0.75		0.30	0.43	0.00				
cBas	62	0.12 ± 0.06	500–600	0.54	0.65	0.62	0.56	0.59	0.56	0.51	0.48	0.56	0.64	0.00			
*G1*	156	0.14 ± 0.09	125–150	0.07	0.14	0.14	0.11	0.16	0.44	0.73	0.69	0.70	0.74	0.56	0.00		
*Gm*	220	0.09 ± 0.05	600–700	0.42	0.57	0.58	0.41	0.52	0.55	0.78	0.77	0.80	0.82	0.70	0.46	0.00	
*G6*	45	0.10 ± 0.05	1250–1500	0.66	0.70	0.69	0.63	0.65	0.64	0.17	0.13	0.34	0.43	0.46	0.62	0.75	0.00

*N* number of accessions; in the cases of *indica* (XI) and *japonica* (GJ), N includes 342 XI-adm and 30 GJ-adm accessions, respectively. *r*^2^: Linkage disequilibrium (LD); ^a^: threshold of pairwise distance between markers above which the mean *r*^2^ reaches values below 0.2. The 64 Admix accessions of the A-panel, the 196 Admix, 1 cAus and 2 cBas accessions of the M-Panel were excluded from LD, gene diversity and genetic differentiation analyses

**Fig. 3:**
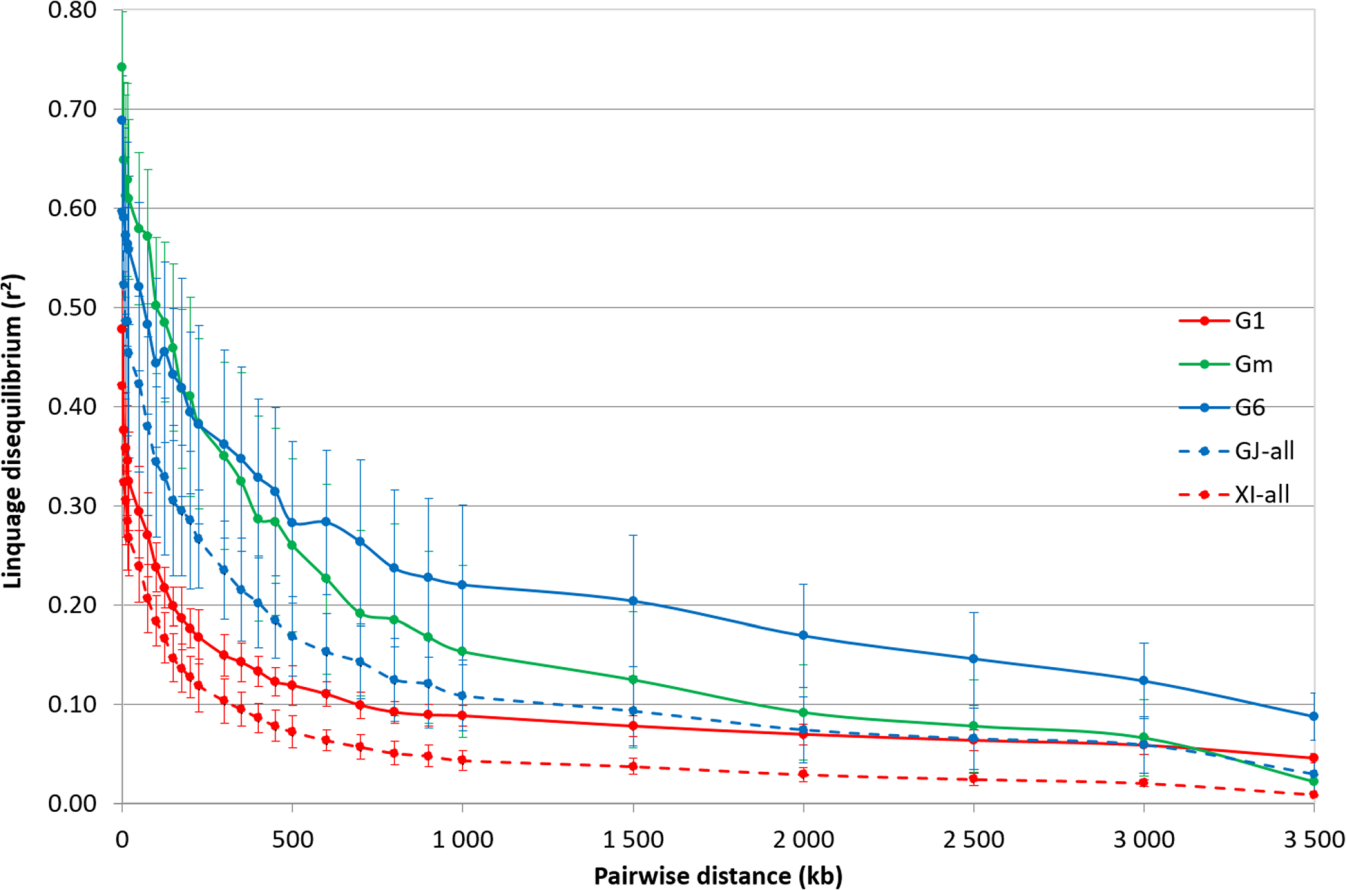
Patterns of decay in linkage disequilibrium in the *indica* and *japonica* population of the Asian panel and the three groups of the Malagasy rice panel, genotyped with 23,981 SNP. The curve represents the average r^2^ according to pairwise distance between markers among the 12 chromosomes and the bars represent the associated standard deviation

Important differences were observed between subpopulations for r^2^ estimate of the initial LD, i.e. pairwise distance between markers below 25 kb (from 0.405 for XI-2 subpopulation to 0.724 for GJ-sbtrop) and for its subsequent decay (Supplementary Table 3). While the distance at which the LD went below 0.2 was between 125 and 150 kb for XI-2, it was above 700 kb for GJ-sbtrop and GJ-temp. Pattern of LD decay in *G1* was similar to the one of the *indica* population but the absolute values were slightly higher (0.05 point) whatever the pairwise distance between markers (Fig. 3). The *G6* group had a high initial LD (r^2^ = 0.594) and rather low rate of LD decay (r^2^ < 0.2 at pairwise distances between markers above 1.5 Mb). The *Gm* group had the highest initial LD (r^2^ = 0.648 for distances below 25 kb) but the LD decay was rather fast (r^2^ < 0.2 at distances above 600 kb) and similar to the one of *G1* for distance above 2 Mb. Compared to *G1* group and Asian subpopulations, *G6* and *Gm* showed larger inter-chromosome variations of LD pattern. For instance, in *Gm*, the average r^2^ values (over 28 levels of pairwise distance between markers) of chromosomes 6 and 11 were + 42% and + 33% higher than in other chromosomes, respectively.

Genetic differentiation between the three Malagasy groups was significant (*p* < 0.05 for 1023 permutations), ranging from 0.46, for *G1-Gm*, to 0.75, for *G6-Gm* (Table 1). However, “among population” variance, calculated by AMOVA, represented only 40% of the total molecular variance (*p* < 0.0001), indicating large within-group differentiation. Differentiations between the Malagasy *G1* and *G6* groups and their Asian counterparts were rather low but highly significant suggesting a specific history of the Malagasy rice varieties. The *G1* - cAus *F*_*ST*_ and the *G1* - cBas *F*_*ST*_ were of the same magnitude as the *indica* - cAus and the *indica* - cBas *F*_*ST*_, respectively. Similar pattern was observed for *G6* - cAus and *G6* - cBas *F*_*ST*_, and its Asian counterpart *japonica*. The *F*_*ST*_ between *Gm* and the Asian subpopulations were also high, ranging from 0.41 to 0.82 (Table 1), confirming the fact that *Gm* could not be directly affiliated to one of those subpopulations. The *F*_*ST*_ between *Gm* and the Asian subpopulations was lowest with XI-2.

### Haplotype Level Genetic Variations

**Fig. 4:**
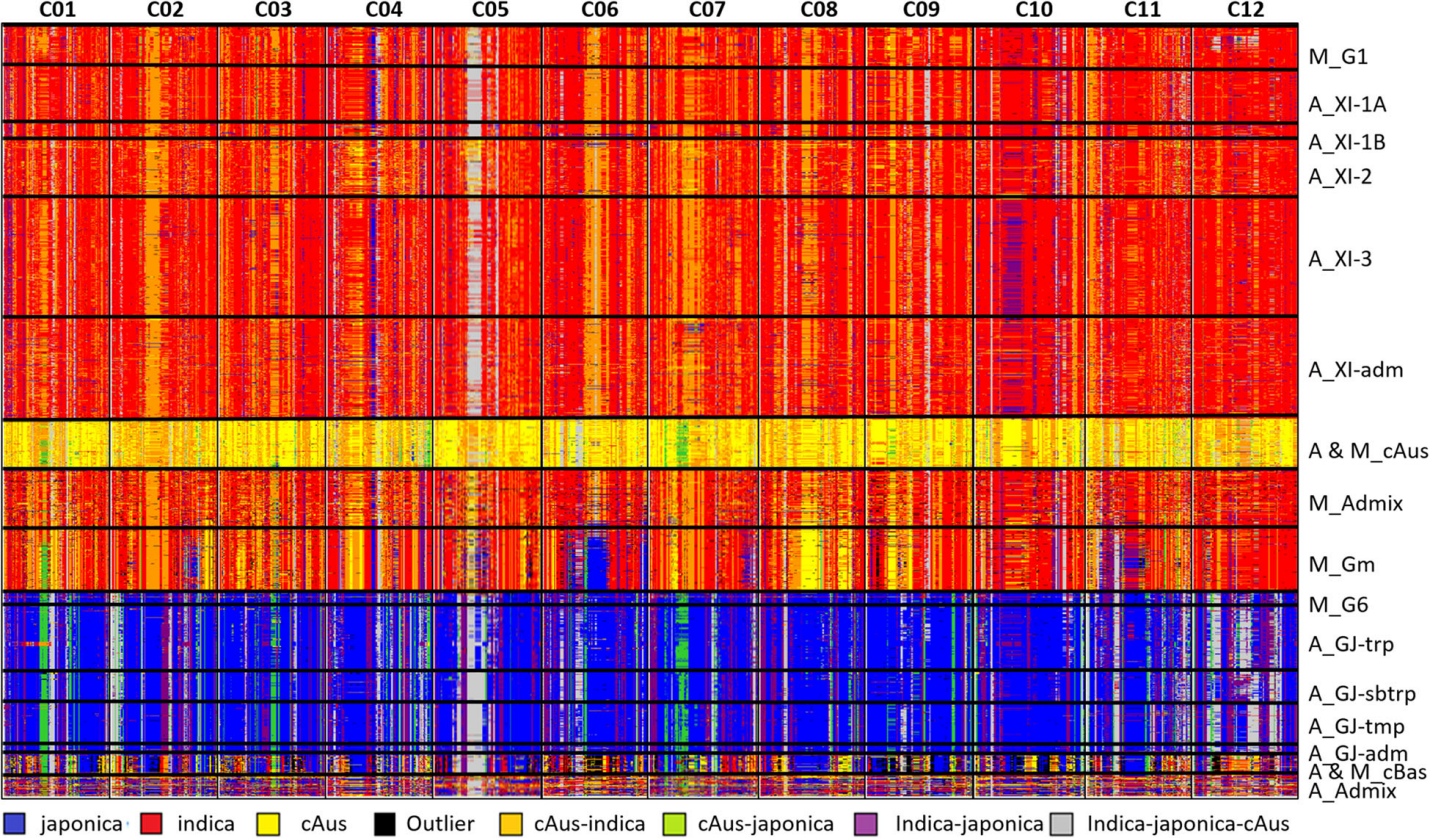
Ideogram of local classification of the genomes of 1929 Asian and 620 Malagasy rice landraces, based on 23,982 SNP. Patterns are organised per chromosome from left to right and by accessions from top to bottom. The Asian accessions (prefix A) are organised first by their classification in Wang et al. (2018) groups and subgroups, then by alphabetic order of country (not shown). Malagasy accessions (prefix M) are organised first by groups identified by structure analysis with K = 3, then by increasing values of ancestry coefficient

Results of the KDE-based local classification are represented for each chromosome in Fig. 4 and are summarised over the whole genome in Table 2. As expected, a large percentage of haplotypes of accessions of the A-panel that were considered as members of the *indica*, *japonica* or cAus subpopulations at the whole genome level (Wang et al.*,* 2018), was attributed by the KDE-based classification to their homologous classes of haplotypes, *ind*, *jap* and cAus. On average, the percentage was of 68.9, 58.4 and 53.1 for *indica*, *japonica* and cAus accessions, respectively. However, variations of up to 35% of these shares existed between chromosomes. The percentages of cross-classification of haplotypes of accessions among the three subpopulations (i.e. *indica* to *jap* and vice-versa, *indica* to cAus and vice-versa, and *japonica* to cAus and vice-versa) were low, rarely exceeding 2%. The percentage of haplotypes attributed to each of the four intermediate classes (*ind*-*jap*, *ind*-cAus, *jap*-cAus, and *ind*-*jap*-cAus) depended on the classification of accessions at the global level. In the case of *indica* accessions, on average 12.3% of the haplotypes were classified as *ind*-cAus and 9% as *ind*-*jap*-cAus, while the average share of the *ind*-*jap* and *jap*-cAus haplotypes were of 4.8% and 2.9%, respectively. In the case of the *japonica* accessions, on average 13.8% of the haplotypes were classified as *ind*-*jap* and 17.7% as *ind*-*jap*-cAus, while the average share of *ind*-cAus and *jap*-cAus haplotypes were of 3.8% and 4.0%, respectively. Accessions belonging to the cAus subpopulation were characterised by a large share of haplotypes (on average 26.4%) classified as *ind*-cAus. The proportions of haplotypes of *indica*, *japonica* and cAus accessions classified as outliers were very low, less than 1%, on average. Accessions of the A-panel belonging to the cBas subpopulation of Wang et al. (2018) were characterised by large shares of *jap* haplotypes, 31.2% on average, and outlier haplotypes, 11.0% on average.

**Table 2:** Mean shares (%) of the eight categories of haplotypes identified by the KDE-based local classification, in each population and subpopulation of the Asian and Malagasy diversity panels

Diversity panel	Global classification	KDE-based local classification							
		*ind*	cAus	*Jap*	*ind*-*jap*	*ind*-cAus	*jap*-cAus	*ind*-*jap*-cAus	Outlier
Asian Panel	**indica**	**68.9**	**1.2**	**0.9**	**4.8**	**12.3**	**2.9**	**8.9**	**0.1**
		(59.8–84.8)	(0.9–1.9)	(0.4–2.7)	(2.1–7.4)	(5.8–19.2)	(1.6–4.5)	(3.8–15.7)	(0–0.4)
	- XI-1	**67.6**	**0.5**	**1.0**	**5.2**	**12.4**	**3.4**	**9.8**	**0.1**
		(57.6–85.6)	(0.2–1.6)	(0.3–2.9)	(2–7.9)	(4.3–17.9)	(1.7–9)	(4.1–18.9)	(0–0.2)
	- XI-2	**65.6**	**2.9**	**0.5**	**4.4**	**14.5**	**2.8**	**8.5**	**0.8**
		(51.4–80)	(1.9–4.8)	(0.2–1.6)	(1.7–6.6)	(8.4–23.2)	(−0.4–4.7)	(1.2–15.1)	(0–8.6)
	- XI-3	**72.7**	**0.3**	**0.5**	**4.6**	**10.9**	**2.7**	**8.3**	**0.1**
		(60.8–88.5)	(0.1–0.7)	(0.1–2.4)	(2–9.3)	(4.1–19.9)	(1.5–4.5)	(3.3–13.9)	(0–0.3)
	- X-adm	**67.2**	**1.5**	**1.4**	**5.0**	**12.6**	**3.0**	**9.2**	**0.1**
		(57.5–83.5)	(1.2–2.3)	(0.7–3.3)	(2.2–7.2)	(6.2–18.9)	(1.7–4.6)	(4.1–16.1)	(0–0.5)
	**japonica**	**1.0**	**0.4**	**58.4**	**13.8**	**3.8**	**4.0**	**17.7**	**0.8**
		(0.4–1.8)	(0.3–0.7)	(36.4–71.6)	(3–23.2)	(2.7–6.5)	(2.7–6.5)	(9.7–27.3)	(0.1–7.2)
	- GJ-trop	**1.1**	**0.5**	**58.2**	**14.7**	**3.7**	**3.9**	**16.0**	**2.0**
		(0.3–2.0)	(0.3–1.1)	(36.3–71.9)	(3.1–23.2)	(1.9–6.4)	(1.8–6.3)	(8.4–27.4)	(0.1–9.8)
	- GJ-sbtrop	**0.8**	**0.4**	**58.4**	**14.5**	**4.1**	**4.2**	**16.5**	**1.3**
		(0.1–1.7)	(0.1–0.9)	(40.9–73.4)	(2.9–23.9)	(2.8–6.9)	(2.8–6.4)	(9.1–25.5)	(0.1–6.6)
	- GJ-temp	**1.0**	**0.2**	**55.7**	**14.6**	**4.5**	**4.6**	**18.2**	**0.8**
		(0.4–2.4)	(0.0–0.6)	(33.7–71.4)	(3.2–23.0)	(2.8–6.9)	(2.9–7.1)	(11.1–28.1)	(0.0–3.9)
	- GJ-adm	**2.7**	**0.7**	**54.8**	**14.8**	**4.4**	**4.4**	**17.1**	**1.5**
		(1.4–5.0)	(0.3–1.1)	(35.3–70.3)	(5.1–22.7)	(3.0–6.5)	(2.8–6.6)	(9.8–27.9)	(0.0–6.6)
	**cAus**	**1.8**	**53.1**	**0.8**	**3.3**	**26.4**	**3.5**	**10.8**	**0.2**
		(1.3–2.3)	(37.6–63.8)	(0.5–2)	(1.7–6.7)	(14.4–42.2)	(1.8–6.7)	(4.8–18.4)	(0–0.4)
	**cBas**	**16.9**	**13.2**	**31.2**	**6.9**	**7.1**	**2.6**	**11.1**	**11.0**
		(10.6–27.4)	(4.9–22.4)	(20.7–38.7)	(2.2–12.9)	(2.9–10.9)	(−0.9–5.4)	(4.4–21.2)	(6.1–23.7)
	**Admix**	**24.4**	**12.4**	**24.3**	**7.4**	**10.6**	**4.3**	**10.3**	**6.2**
		(20.1–27.2)	(8.7–16.4)	(16.4–30.9)	(2.3–11.3)	(7.5–14.5)	(2–13.3)	(1.8–17.2)	(4.9–9.7)
Malagasy panel	***G1***	**69.7**	**1.3**	**0.6**	**5.0**	**11.9**	**2.8**	**8.4**	**0.4**
		(59.9–84.2)	(0.6–2.3)	(0.1–2.1)	(2.0–7.6)	(4.5–17)	(1.6–4.2)	(3–16.2)	(0.1–1.2)
	***G6***	**2.7**	**2.0**	**57.4**	**12.5**	**3.9**	**3.8**	**15.0**	**3.1**
		(1.5–4.8)	(0.8–4.1)	(38.3–69.0)	(3.0–18.9)	(2–7)	(1.6–6.8)	(7.1–23.1)	(1.2–10.8)
	***Gm***	**50.6**	**8.2**	**5.4**	**5.7**	**14.3**	**3.3**	**9.8**	**2.6**
		(34.9–79.6)	(2.4–19.8)	(1.2–24)	(2.1–13.2)	(6–21.5)	(1.8–6.3)	(3.7–19.2)	(1.3–3.8)
	**Admix**	**58.9**	**6.2**	**3.4**	**4.6**	**12.5**	**2.8**	**7.4**	**4.1**
		(49.2–75.0)	(2.4–8.3)	(2.6–5.1)	(1.9–8.8)	(5.0–20.5)	(1.0–5.2)	(1.6–12.9)	(2.3–6.2)

Among accessions of the M-Panel, the shares of the eight classes of haplotypes in the *G1* group were similar to the ones observed for its Asian counterpart, *indica*. In the case of *G6* the shares differed slightly from its Asian counterpart, *japonica*, with higher percentages of haplotypes classified as *ind* and cAus. Patterns of distribution of the eight classes of haplotypes among the accessions of *Gm* group, significantly diverged from the ones observed for all other groups. It displayed a significantly lower share of *ind* haplotypes (50.6% on average) and significantly higher shares of cAus, *jap* and outlier haplotypes than the Asian *indica* and the Malagasy *G1* group, 8.2%, 5.4% and 2.6% on average, respectively. The Malagasy Admix had a haplotype pattern similar to *Gm*, with slightly lower shares of cAus and *jap* haplotypes.

To further investigate the relationship between the *Gm* and the Asian *indica*, we compared the patterns of distribution of haplotypes of *Gm* accessions with the ones of each of the four *indica* subpopulations, XI-1, XI-2, XI-3 and XI-adm (Wang et al., 2018). Patterns of distribution of eight classes of haplotypes in the *Gm* group diverged from the ones of the four *indica* subpopulations by lower share of *ind* haplotypes and higher shares of cAus and *jap* haplotypes. The divergence was the lowest with the XI-2 subpopulation.

The *Gm* accessions also displayed larger variations of the shares of each class of haplotype across the 12 chromosomes. For instance, chromosome 6 displayed an exceptionally high share of *jap* haplotypes (24% on average), chromosome 8 a large share of cAus haplotypes (19.8% on average) and chromosome 11 a low share of *ind* and high shares of *ind*-*jap* and *ind*-*jap*-cAus haplotypes, 34.9%, 13.2% and 19.2%, respectively. In some cases, these haplotypes were contiguous and covered a large segment of the chromosome. For instance, on chromosome 6, one of the *jap* haplotype segments spread over 4.7 Mb (13.0 to 17.7 Mb) and on chromosome 8 one of the cAus haplotype segments spread over 5.4 Mb (10.9–16.3 Mb).

Dissymmetry based neighbour-joining tree constructed with the genotypic data of individual chromosomes (Supplementary figure 4) confirmed the heterogeneity of shares of different classes of haplotype among the 12 chromosomes. It also showed the discriminatory power of genetic diversity at the individual chromosome level. Accessions belonging to *Gm* were always clustering in a well-individualised branch of the tree, often near the cAus branch. M-Admix accessions almost systematically clustered with the *Gm* accessions. The chromosome level neighbour-joining trees also allowed, in some cases, to identify accessions of the M-panel and A-panel that displayed similar patterns of haplotypes shares. In the case of the M-panel, the three accessions most often clustering with *Gm* belonged to *G1* originated from the Mahajanga region on the west coast of Madagascar. In the case of the A-panel, more than 95% of accessions clustering near *Gm* were of Indian subcontinental origin and belonged to XI-2 subgroup (66%), XI-adm (21%) and cAus (13%). Three accessions of the A-panel most frequently present in the vicinity of the *Gm* cluster were of Indian origin: Larha Mugad (IRGC 52339–1) from Karnataka, Adukkan (IRGC 81783–1) from Kerala and Cuttack 29 (IRGC 49573–1) from Odisha. Larha Mugad and Adukkan belonged to XI-2, Cuttack 29 was an XI-adm.

### Characteristics of the Large cAus and *Jap* Chromosomic Segments in the *Gm* Group

The two largest *jap* and the largest cAus chromosomic segments in the *Gm* accessions were investigated for kinship with their homologous segments in other Malagasy groups and in Asian subpopulations, and for their functional proprieties. Dissimilarity based phylogenetic neighbour-joining trees were constructed using the genotypic data from each of the three segments (240, 221 and 225 SNP loci for chromosome 6, 8 and 11, respectively) for kinship analysis. The functional properties of the three large introgression segments was investigated through gene ontology analysis (Mi et al. 2013) and compilation of results of ten recent genome-wide association studies (GWAS) for response to low temperature stress, between 2015 and 2020.

**Fig. 5:**
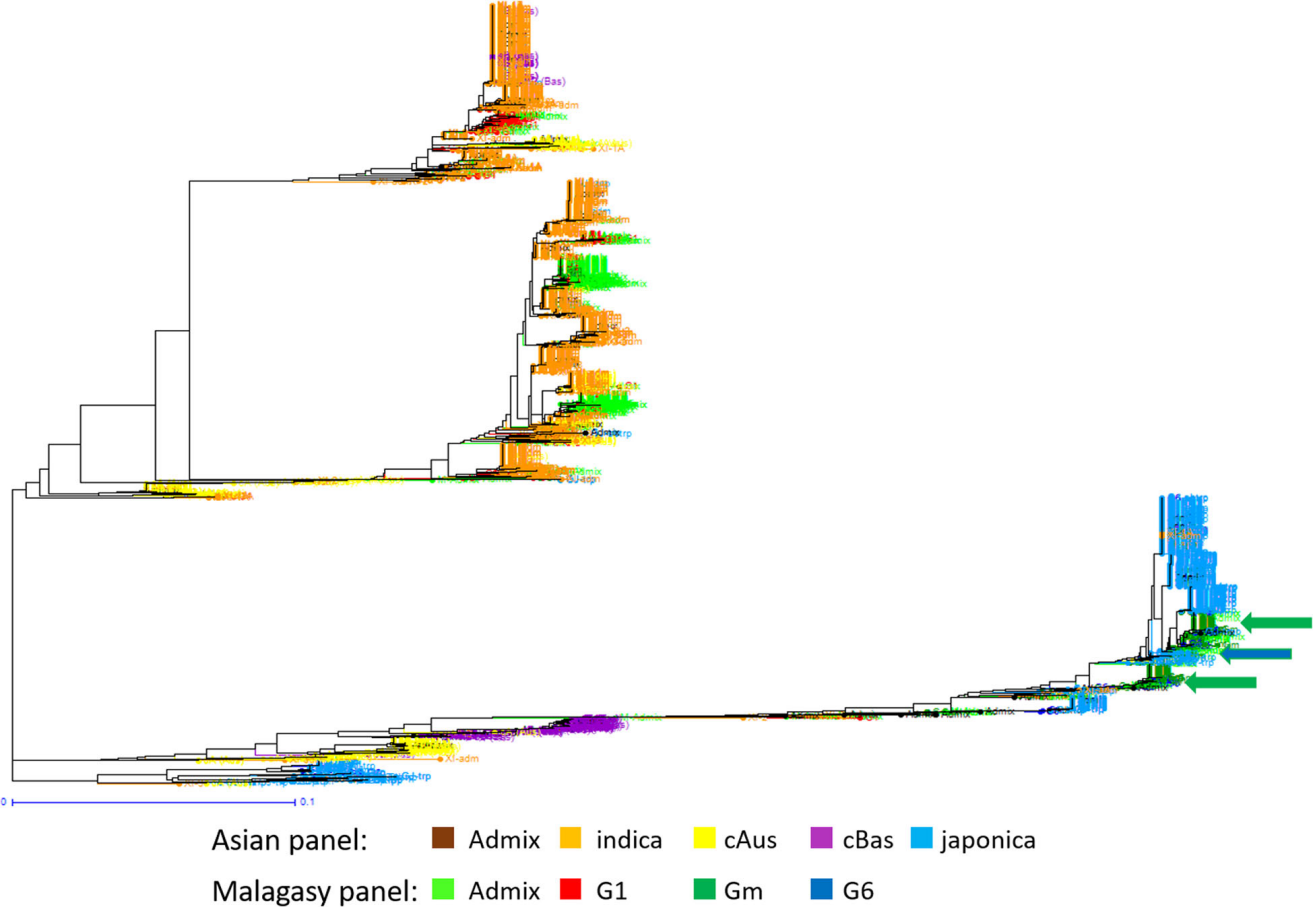
Unweighted neighbour-joining tree of simple matching distances, constructed with the genotypic data at 13.0–17.7 Mb segment of chromosome 6 (240 SNP). Accessions of the Asian panel are coloured according to their membership to major populations defined by Wang et al. (2018). Accessions of the Malagasy panel are coloured according to their membership of sNMF groups at K = 3 and ancestry coefficient threshold of 0.8. Positions of G6 and Gm accessions on the trees are highlighted with coloured arrows corresponding to their group membership

In the neighbour-joining trees constructed with the genotypic data from the 13.0 to 17.7 Mb segment of chromosome 6, the *Gm* accessions and a significant share of the Malagasy Admix accessions formed a rather compact branch in the vicinity of the *japonica* cluster (Supplementary figure 5). *Japonica* accessions most closely clustering with *Gm* accessions were members of the Malagasy *G6* group, (Fig. 5). The non-Malagasy accessions clustering near *Gm* were GJ-trop from Indonesia, Malaysia and the Philippines, plus one Admix accession from India.

In the neighbour-joining trees constructed with the genotypic data from the 10.6 to 15.7 Mb segment of chromosome 11, *Gm* accessions formed again a rather compact branch near the *japonica* cluster. *Japonica* accessions most closely clustering with *Gm* belonged to *G6*, to GJ-trop from Indonesia and, to a lesser extent, to GJ-sbtrop from India and GJ-tem from China (Supplementary figure 5).

In the neighbour-joining trees constructed with the genotypic data from the 10.9–16.3 Mb segment of chromosome 8, *Gm* accessions formed a compact cluster near the cAus subpopulation. Two cAus accessions from Bangladesh and India were embedded in the *Gm* cluster. The closest neighbours to the *Gm* cluster were 30 cAus, 4 XI-2 and one XI-adm accessions of Bangladeshi and Indian origin (Supplementary figure 6).

Gene ontology analysis (Supplementary Table 4) detected highly significant (78.5 fold) enrichment in “cold acclimation” genes in the *japonica* introgression of chromosome 11. It also detected small but significant enrichment among the unclassified genes of the three introgression segments. Compilation of results GWAS for response to low temperatures led to 610 quantitative trait loci (QTL) of which 22, 11 and 8 were located in the introgression segment of chromosomes 6, 8 and 11 respectively (Supplementary Table 5). These numbers were on average of 1.35 QTL per Mb of genome length outside the introgression segments and on average 4.63, 2.02 and 1.56 QTL per Mb, in the introgression segment of chromosomes 6, 8 and 11 respectively. Lastly, a literature review for gene families involved in cold tolerance and response to abiotic stresses, identified 29, 25 and 14 genes with possible involvement in response to low temperatures, in the introgression segment of chromosomes 6, 8 and 11 respectively (Supplementary Table 6).

## Discussion

The objective of this work was to use the power of high-resolution genotypic data to gain new insights into the organisation of rice genetic diversity in Madagascar and, beyond, into the evolutionary processes accompanying *O. sativa*’s geographical expansion.

The population structure of rice in Madagascar, with its three major groups *G1*, *G6* and *Gm*, revealed in this study corresponded to the large scale Asian differentiation of *O. sativa*, but with two significant differences: (i) only the two major Asian subpopulations, *indica* and *japonica* were significantly represented; the cBas and cAus subpopulations were each represented by two accessions only. (ii) Madagascar hosted an additional group (*Gm*) that could not be assigned to any of the four major Asian subpopulations. The existence of an atypical rice group was first reported by Ahmadi et al. (1988), using morpho-physiological data, and then confirmed using isozyme (Ahmadi et al., 1991) and SSR markers (Radanielina et al. 2012). However, so far, the *Gm* group has not been identified as such in studies directly comparing Malagasy varieties with a diversity panel originating from Asia. Indeed, Mather et al. (2010), who compared 45 Malagasy landraces with 39 Asian accessions (21 *indica* and 18 GJ-trop), identified only one group among the Malagasy lowland accessions and called it Malagasy-*indica*. Zhao et al. (2011), who genotyped a rice diversity panel of 415 accessions with 44 K SNP, classified the 6 Malagasy accessions present as admixed and not as belonging to one of the four major Asian subpopulations. Thus, our study is the first one comparing a Malagasy and an Asian diversity panel on a large enough scale to distinguish the *Gm* group from both Malagasy and Asian originating *indica* subpopulations. The Malagasy-*indica* of Mather et al. (2010) most likely corresponded to our *Gm* group.

Gene diversity in *G1* and *G6* represented only 75% and 66% of the diversity in the Asian *indica* and *japonica*, respectively. This reduced diversity represents the bottleneck associated with the introduction of rice to Madagascar from different Asian sources. Consistent with the relative diversity of Asian and Malagasy-*indica* reported by Mather et al. (2010), the gene diversity of *Gm* represents 51% of that of *indica*.

**Fig. 6:**
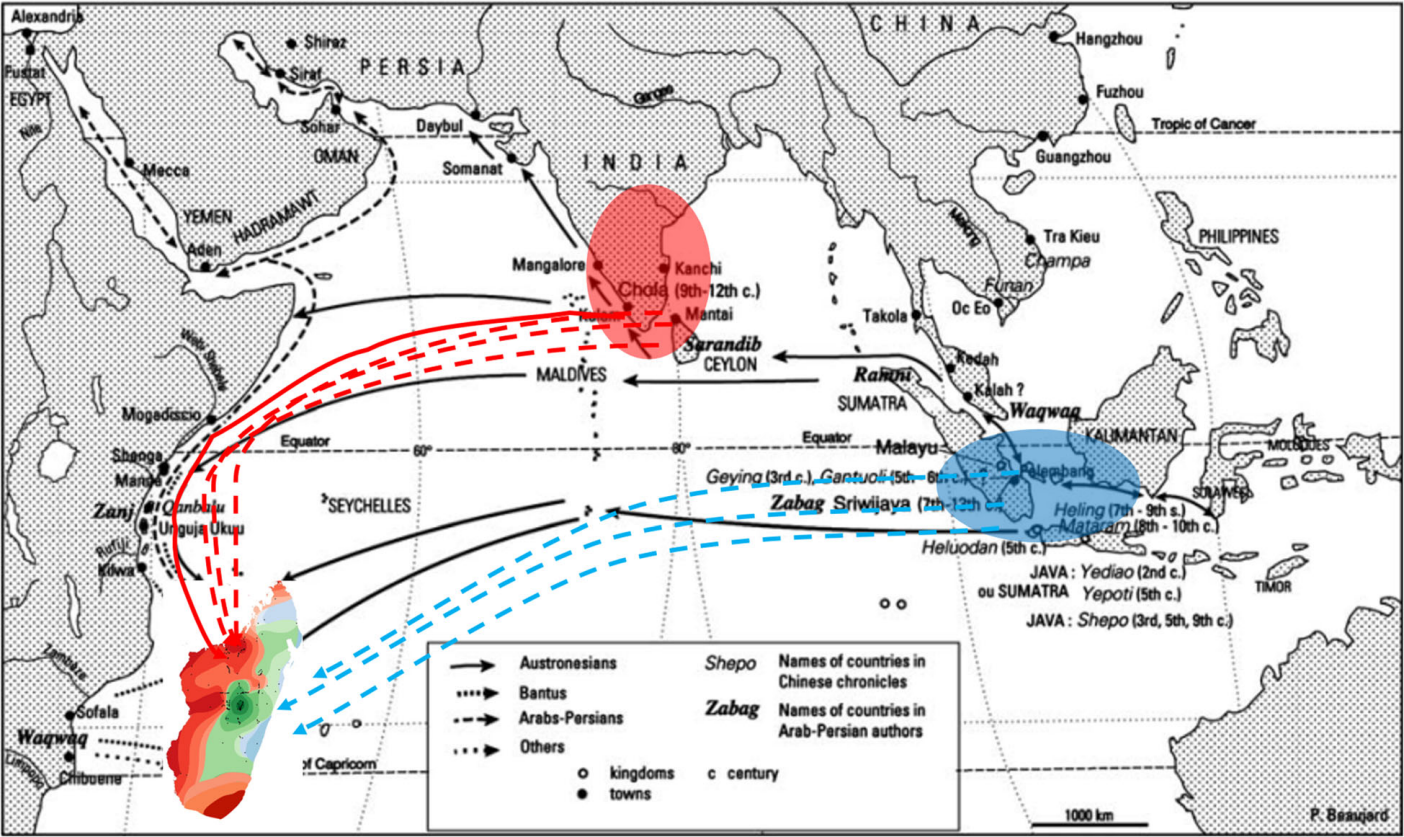
History of human migrations to Madagascar, the associated introductions of different rice genetic groups, and the current eco-geographical distribution of rice groups in the country. The map of human migrations (black arrows) was borrowed from Beaujard (2011). The red and blue circles indicate the most probable area of origin of the Malagasy *indica* and *japonica* rice groups, respectively. The blue dotted lines indicate the arrival in Madagascar of the *japonica* rice varieties, associated with the South-westward migration of population of Malay origin (1200–1400 AD). The red dotted lines indicate the arrival of the *indica* rice varieties associated with the waves of Austronesians reaching Madagascar up to the fifteenth century AD. The red bold line represents the latest wave of Austronesians migrants who established the Merina kingdom in the central highlands of the country and developed rice cultivation in those areas. The red, blue and green colours covering the Malagasy island indicate du current eco-geographical distribution of rice groups in the country

The three Malagasy groups are not evenly distributed across the country. One major factor shaping this uneven geographic distribution is the climatic conditions associated with altitude, leading to a preferential habitat for each group sketched in Fig. 6. Similar to the area of cultivation of *indica* in tropical Asia and elsewhere in the world, *G1* accessions are mainly cultivated in the low-elevation coastal wetlands. The preferential habitat of *Gm* is the wetlands of 1200-1700 m elevation, while in tropical Asia such areas are mainly occupied by GJ-temp. The eco-geographic distribution of *G6* is more complex, some accessions being cultivated in the upland ecosystem of the Eastern-forests areas and some others in the lowland ecosystem of the central high plateaux.

The structuring of rice genetic diversity in three major groups and their uneven eco-geographical distribution in Madagascar is also related to the history of the introduction of rice in the island summarised in Fig. 6. The tight clustering of *G6* accessions with the GJ-trop accessions originating from Indonesia on the neighbour-joining tree corroborates historical and linguistic data of the Austronesian (present Malaysia and Indonesia) origin of the human population that first settled the Madagascar island 1400–1200 years ago (Dahl, 1951; Tofanelli et al., 2009; Cox et al., 2013). The swidden cultivation of upland rice along the east coast of Madagascar started with these first colonisations of the island (Beaujard, 2011). The Malagasy lowland *japonica* varieties (“*Vary Lava*” and “*Latsika*” vernacular families, well known for very long and thick grains, and for cold tolerance, respectively) also belong to the GJ-trop subpopulation. Varieties belonging to the *Bulu* ecotype of Indonesia are the archetypes of lowland-grown GJ-trop (Glaszmann, 1987; Thomson et al. 2007). Some examples have also been reported in the Philippines, with varieties such as Azucena. The presence of lowland grown GJ-trop in Madagascar is thus another clue backtracking the origin of the Malagasy *japonica* varieties to Indonesia. Interestingly almost all Indonesian lowland-grown landraces of GJ-trop genotype originated from Central Java, Kalimantan and Sumatra islands (Thomson et al., 2007) where more than 500,000 ha of rice are grown in terraced fields at an altitude above 700 m (Harahap, 1979). In these areas, typical *indica* varieties such as IR8 yield very poorly and cold-tolerant varieties are needed (Harahap, 1979). As the cold tolerance of the GJ-temp is generally associated with round grain type, the Malagasy long grain “*Vary Lava*” varieties constitute a useful source for the diversification of grain quality in breeding for temperate regions and cold-prone tropical areas.

The characteristics of the Malagasy *G6* group also provide insight into the evolutionary process that accompanied *japonica* subspecies’ expansion. First domesticated in the Yangzi region of China (Liu et al.*,* 2007; Molina et al., 2011), the *japonica* subspecies has accompanied the southward migrations of human populations reaching Taiwan by 3000 BC and Central Indonesia by 2000 BC (Bellwood, 2011). When the *japonica* subspecies began its migration south-westward to Madagascar more than 2500 years later (in the middle of the first millennium AD), it had already reached its current balance of recent and ancestral alleles, as shown by the almost equal mean rate of *jap* haplotypes in *G6* compared to the *japonica* subpopulation. On the other hand, compared to *japonica*, *G6* showed a slightly higher mean rate of *ind* and c*Aus* haplotypes, suggesting some genetic expansion during the south-westward waves of migration or after insulation in Madagascar. Given the migration route of the early Austronesian settlers, the latter option seems the most probable. Whatever the origin of the genetic expansion, it has endowed the *G6* group with enough diversity to allow adaptation to both the warm upland ecosystem of East coast-forest areas and the cold lowland ecosystem of the high plateaux.

According to historical and linguistic data (Boiteau, 1977; Domenichini-Ramiaramanana 1988; Allibert, 2007; Beaujard, 2011), waves of Austronesians reaching Madagascar went on until the fifteenth century AD, using more complex routes and borrowing Dravidian and Arabo-Persian linguistic, cultural and rice cropping practices on their way. While the lowland rice farming vocabulary of Malagasy originates in the Malay Archipelago, yet terms from Indian, Arabic and Swahili origins are also frequent, especially on the west coast and in the central high plateaux (Beaujard, 2003, 2011). These data are consistent with our finding of preferential clustering of a large share of *G1* accessions with accessions of the XI-2 subpopulation of *indica* originating from Bangladesh, India and Sri-Lanka. A tight relationship between Madagascar and Indian subcontinent *indica* accessions was also reported by Mather et al. (2010), based on the clustering of some of their Indian *indica* accessions with the Malagasy-*indica* group.

The latest arrivals of Austronesians, who disembarked on the west coast and later founded the Merina kingdom in the central high plateaux, are believed to have played an important role in the development of lowland rice cultivation in Madagascar (Boiteau, 1977; Beaujard, 2011). The establishment of the *Gm* group in the high plateaux is probably associated with this last major immigration wave. Indeed, rice cultivation was absent from the high plateaux before the establishment of the Merina ethnic group (Raison, 1972; Abé, 1984) and the contemporary preferential area of distribution of the *Gm* group coincides with the area of establishment of the Merina and their Betsileo cousins.

Accessions of the *Gm* group are characterised by a low mean share of *ind* haplotypes, down to 35% in chromosome 11, and high mean shares of cAus and *jap* haplotypes, up to 20% and 24%, in chromosome 8 and 6, respectively. Within the A-panel, apart from cAus per se, such high share of cAus haplotypes is observed only in some cBas accessions. Given the almost absence of representatives of c*Aus* subpopulation in Madagascar, the most parsimonious hypothesis regarding the origin of the cAus haplotypes in *Gm* group is a recombination between *indica* and cAus in the Asian area of origin. Analysis of the origin of the large segments of chromosomes 1 and 8 composed of cAus haplotypes in *Gm* group indicates a high proximity with two cAus, four XI-2 and one XI-adm accessions of Bangladeshi and Indian origin. Among these accessions at least three [Mugad (IRGC 52339–1), Adukkan (IRGC 81783–1), and Cuttack 29 (IRGC 49573–1)] are from the East (Odisha) and South-West (Kerala, Karnataka) coastal states of India, situated along the maritime roads of the latest Austronesians’ migrations to Madagascar, described by Beaujard (2011). Interestingly, (i) western Odisha is recognized as part of the center of origin of *Aus* ecotypes (Sharma et al. 2000). (ii) Glaszmann (1988) reported the presence of *indica*, *Aus, Aromatic* and *intermediate* accessions among the primitive cultivars of the Jeypore tract of Odisha, thought to have been isolated from alien rice and thus representative of local domestication processes. (iii) The Odisha State includes rice growing areas at elevation above 750 m on the one hand and dry season rice cultivation practices with temperature below 18 °C (Das, 2012), on the other hand.

Regarding the origin of the high mean share of *jap* haplotypes in *Gm* accessions, its occurrence in all chromosomes (with larger proportions in chromosomes 6, 7 and 11) with complex patterns, sometimes corresponding to very unlikely double or triple crossing-overs, argues for multiple and rather ancient hybridisation events resulting from sympatry between GJ-trop accessions and *indica*-cAus intermediate forms. The rarity of representatives of the GJ-trop subpopulation in the rims of the Indian subcontinent, and the rarity of representatives of *indica*-cAus intermediate forms in the Malo-Indonesian archipelago, excludes the possibility of their sympatry in these places. Quite the reverse, the high plateaux of Madagascar have been hosting representatives of the two subpopulations for some five centuries. Interestingly, a large majority of *G6* accessions most closely clustering with the *Gm* group in the neighbour-joining tree constructed with genotypic data from the large *jap* haplotypes of chromosomes 6 and 11, originates from the high plateaux of Madagascar (collection mean altitude of 1240 m) and is cultivated in the lowland ecosystem. The presence in Madagascar of a large number of Admix accessions, that cluster with *Gm* when cAus haplotypes are considered and differ from *Gm* for the share of *jap* haplotypes (mean share of 3.4% per chromosome against 5.4% for *Gm*), reinforces the hypothesis of a local, Malagasy, origin of *Gm* group. It emerged from multiple hybridisation and recombination between *indica*-cAus intermediary forms and *japonica* (*G6*) groups. The footprint of the most recent such hybridisations is still visible in chromosomes 6 and 11 of *G6*, with much slower LD decay. The hypothesis of *Gm* emergence from local hybridisation is also in agreement with the high rate of fertility (reaching 70%) of F1 hybrids of *indica* x *Bulu* crosses (Morinaga and Kuriyama, 1958). However, as the usual fate of the recombinant progenies of such crosses is a replacement by parental forms (Oka, 1983), their survival and expansion is most likely due to some forms of positive selection. The preferential eco-geographic distribution of *Gm* accessions in the high plateaux of Madagascar (1200–1600 m), argues for the selection for cold tolerance.

Mather et al. (2010) also reported evidence of several separate *indica* x *japonica* hybridisations and recombinations in Madagascar. The extent of the *Gm* group’s gene diversity revealed in the present study (equivalent to the one of GJ-trop subpopulation and much larger than the ones of GJ-sbtrop and GJ-temp), and the pattern of its LD decay (faster than the ones of the three subpopulations of *japonica*) argue for its recognition as an original group. The recognition as an original group, instead of “Admix”, would be helpful for its future utilisation as a valuable genetic resource for adaptation to high elevation areas. Indeed, “Admix” accessions are seldom included in genetic studies or used in breeding programs. We propose the name “*Rojo*” for this original rice group. This prefix is shared by almost 25% of names of the accessions belonging to *Gm* and refers to their grain shape associating a medium-long length with a large width (Peltier 1970; Ahmadi et al. 1988).

Despite the complex underlying evolutionary and demographic events in the making of the Malagasy rice gene pool, we were able to backtrack different components of the *O. sativa* diversity present in Madagascar to their Asian geographic area of origin and genetic subpopulations and to confirm the presence of the original *Gm* group. Thanks to the multiplicity of introduction events borrowing several routes, the bottleneck undergone by *G1* and *G6* groups has not been too severe. The evolutionary process involved in the emergence and expansion of the *Gm* ecotype, are most probably (i) multiple hybridisations and recombinations between *indica* and cAus subpopulations in the context of their sympatry in south Asia. (ii) Several hybridisations and recombinations between representatives of *indica*-cAus admixes and *G6* group in the context of sympatry in the high plateaux of Madagascar. (iii) Human selection for adaptation to the lowland rice cultivation in the high plateaux. The rather large genetic diversity of the *Gm* group, the existence of intermediaries or admixed forms between *G1* and *Gm*, and the complementary preferential habitats of *G1* and *Gm* in terms of altitude and associated climatic conditions, provide a unique opportunity to investigate rice adaptation to thermal aspects of climate changes, using landscape genomic approach.

## Supplementary Information

**Additional file 1: Supplementary Figure S1**: Heat map of distribution of the two set SNP loci (32,614 and 23,981) along the genome.

**Additional file 2: Supplementary Figure S2**: Population structure in 1929 accessions of the Asian panel estimated from 23,981 genome wide SNPs. The subpopulations are coloured according to their membership to groups defined by Wang et al. (2018).

**Additional file 3: Supplementary Figure S3:** Altitudinal distribution of the Malagasy panel 620 rice accessions according to their membership to groups defined by the sNMF-based analysis of population structure.

**Additional file 4: Supplementary Figure S4**: Unweighted neighbour-joining tree of simple matching distances, constructed with the genotypic data of individual chromosomes (C1 to C12). Accessions of the Asian panel are coloured according to their membership to subpopulations defined by Wang et al. (2018). Accessions of the Malagasy panel are coloured according to their membership to sNMF groups at K=3 and ancestry coefficient threshold of 0.8. Positions of the Malagasy accessions on the trees are highlighted with coloured arrows corresponding to their group membership. Accessions of the Asian panel are coloured according to their membership to major populations defined by Wang et al. (2018).

**Additional file 5: Supplementary Figure S5**: Unweighted neighbour-joining tree of simple matching distances, constructed with the genotypic data at 11.6 – 15.7 Mb segment of chromosome 11 (225 SNP). Accessions of the Asian panel are coloured according to their membership to major populations defined by Wang et al. (2018). Accessions of the Malagasy panel are coloured according to their membership to sNMF groups at K = 3 and ancestry coefficient threshold of 0.8.

**Additional file 6: Supplementary Figure S6**: Unweighted neighbour-joining tree of simple matching distances, constructed with the genotypic data at 10.9 – 16.3 Mb segment of chromosome 8 (221 SNP). Accessions of the Asian panel are coloured according to their membership to major populations defined by Wang et al. (2018). Accessions of the Malagasy panel are coloured according to their membership to sNMF groups at K = 3 and ancestry coefficient threshold of 0.8.

**Additional file 7: Supplementary Table S1**: List and main characteristics of the 620 Malagasy rice accessions used in the present study.

**Additional file 8: Supplementary Table S2**: List of the 3K-RG project accessions used in the present study.

**Additional file 9: Supplementary Table S3:** Decay of pairwise linkage disequilibrium with distance between markers, among the three groups of the Malagasy panel and in seven subpopulations of the Asian panel.

**Additional file 10: Supplementary Table S4:** GO enrichment analysis in the three in introgression segments of Gm group, using tool proposed at http://geneontology.org/.

**Additional file 11: Supplementary Table S5:** Compilation of results of ten genome wide association studies for response to low temperature stress in rice.

**Additional file 12: Supplementary Table S6:** list of genes in the three in introgression segments of Gm group, with possible connection with response to low temperature.

## Data Availability

The genotypic datasets generated and analysed during the current study are available in hapmap format at the following address http://tropgenedb.cirad.fr/tropgene/JSP/interface.jsp?module=RICE, in the tab “STUDIES” then selecting for “Genotypes” in *Study type* and for “GS-Ruse_IRRI-Reference-Population.

## Abbreviations

3 K RGP: 3000: rice genomes project
cAus: Genetic group including the Aus ecotype
cBas: Genetic group including the Basmati ecotype
Fst: Genetic differentiation
G1: Genetic group representative of the indica subspecies in Madagascar
G6: Genetic group representative of the japonica subspecies in Madagascar
GJ: Japonica subspecies
GJ-adm: Admixed japonica
GJ-sbtrop: Subtropical japonica
GJ-temp: Temperate japonica
GJ-trop: Tropical japonica
Gm: Genetic group specific to Madagascar
KDE: Kernel density estimation
LD: Linkage disequilibrium
MAF: Minor allele frequency
PCA: Principal component analysis
sNMF: Sparse nonnegative matrix factorization
SNP: Single nucleotide polymorphism
XI: Indica subspecies
XI-1A: Indica subpopulation mainly originated from East Asia
XI-1B: Indica subpopulation of divers origine
XI-2: Indica subpopulation mainly originated from South Asia
XI-3: Indica subpopulation mainly originated from Southeast Asia
XI-adm: Admixed indica

## References

[CR1] 3K-RGP (2014). The 3,000 rice genomes project. GigaScience.

[CR2] Abé Y (1984). Le riz et la riziculture à Madagascar, une étude sur le complexe rizicole d’Imerina.

[CR3] Ahmadi N, Becquer T, Larroque C, Arnaud M (1988). Variabilité génétique du riz (*Oryza sativa* L.) à Madagascar. L'agronomie tropicale.

[CR4] Ahmadi N, Glaszmann JC, Rabary E (1991). Traditional highland Rices originating from intersubspecific recombination in Madagascar. Rice genetics II.

[CR5] Alexander DH, Lange K (2011). Enhancements to the ADMIXTURE algorithm for individual ancestry estimation. BMC Bioinformatics.

[CR6] Allibert C (2007). Migration austronésienne et mise en place de la civilisation malgache. Lectures croisées: linguistique, archéologie, génétique, anthropologie culturelle. Diogène.

[CR7] Beaujard P (2003). Les arrivées austronésiennes à Madagascar: vagues ou continuum?. Etudes Océan Indien.

[CR8] Beaujard P (2011). The first migrants to Madagascar and their introduction of plants: linguistic and ethnological evidence. Archaeological Res Africa.

[CR9] Bellwood P (2011). The checkered prehistory of rice movement southwards as a domesticated cereal from the Yangzi to the equator. Rice.

[CR10] Boiteau P (1977). Les protomalgaches et la domestication des plantes. Bull Acad Malgache.

[CR11] Bradbury PJ, Zhang Z, Kroon DE, Casstevens TM, Ramdoss Y, Buckler ES (2007). TASSEL: software for association mapping of complex traits in diverse samples. Bioinformatics.

[CR12] Cheng C, Motohashi R, Tsuchimoto S, Fukuta Y, Ohtsubo H, Ohtsubo E (2003). Polyphyletic origin of cultivated rice: based on the interspersion pattern of SINEs. Mol Bio Evol.

[CR13] Civán P, Craig H, Cox CJ, Brown AT (2015). Three geographically separate domestications of Asian rice. Nat Plants.

[CR14] Cox MP, Nelson MG, Tumonggor MK, Ricaut FX, Sudoyo H (2013). A small cohort of island southeast Asian women founded Madagascar. Proc R Soc B.

[CR15] Dahl O (1951). Malgache et Maanyan.

[CR16] Das SR (2012). Rice in Odisha. IRRI technical bulletin no. 16.

[CR17] Domenichini-Ramiaramanana B, Revel N (1988). Madagascar. *Le riz en Asie du Sud-Est. Atlas du vocabulaire de la plante*.

[CR18] FAO (1996) The State of the World's Plant Genetic Resources for Food and Agriculture. http://www.fao.org/agriculture/crops/core-themes/theme/seeds-pgr/sow/sow2/country-reports/en/. Countries reports, Madagascar

[CR19] Frichot E, François O (2015). LEA: AnR package for landscape and ecological association studies. Methods Ecol Evol.

[CR20] Frichot E, Mathieu F, Trouillon T, Bouchard G, François O (2014). Fast and efficient estimation of individual ancestry coefficients. Genetics.

[CR21] Garris AJ, Tai TH, Coburn J, Kresovich S, McCouch S (2005). Genetic structure and diversity in *Oryza sativa* L. Genetics.

[CR22] Glasmann JC (1988). Geographic pattern of variation among Asian native rice cultivars (Orysa sativa L.) bases on fifteen isozyme loci. Genome.

[CR23] Glaszmann JC (1987). Isozymes and classification of Asian rice varieties. Theor Appl Genet.

[CR24] Harahap Z (1979). Rice improvement for cold-tolerance in Indonesia. Report of rice cold tolerance workshop.

[CR25] Higham C, Lu TLD (1998). The origins and dispersal of rice cultivation. Antiquity.

[CR26] Huang X, Kurata N, Wei X, Wang Z, Wang A (2012). A map of rice genome variation reveals the origin of cultivated rice. Nature.

[CR27] Jain S, Jain R, McCouch S (2004). Genetic analysis of Indian aromatic and quality rice (Oryza sativa L.) germplasm using panels of fluorescently-labelled microsatellite markers. Theor Appl Genet.

[CR28] Kovach MJ, Calingacion MN, Fitzgerald MA, McCouch SR (2009) The origin and evolution of fragrance in rice (*Oryza sativa* L) *PNAS* 106: 14444–14449, 34, DOI: 10.1073/pnas.0904077106 10.1073/pnas.0904077106 PMC2732888 19706531

[CR29] Liu L, Lee G, Jiang L, Zhang J (2007). Evidence for the early beginning (c.9000 cal. BP) of rice domestication in China: a response. Holocene.

[CR30] Ma J, Bennetzen JL (2004). Rapid recent growth and divergence of rice nuclear genomes. PNAS.

[CR31] Maclean JL, Dawe DC, Hardy B, Hettel GP (2002) Rice almanac. Source book for the most important economic activity on earth. IRRI. DAPO box 7777, metro Manila, Philippines

[CR32] Mansueto L, Fuentes RR, Borja FN, Detras J, Abrio-Santos JM (2017). Rice SNP-seek database update: new SNPs, indels, and queries. Nucleic Acids Res.

[CR33] Mather KA, Molina J, Flowers JM, Rubinstein S, Rauh BL (2010). Migration, isolation and hybridization in island crop populations: the case of Madagascar rice. Mol Ecol.

[CR34] McKinney W (2012) Python for data analysis: data wrangling with Pandas, NumPy, and Python. O’Reilly Media, Inc. https://www.oreilly.com/.

[CR35] Mi H, Muruganujan A, Casagrande JT, Thomas PD (2013) Large-scale gene function analysis with PANTHER Classification System. Nat Protoc 8(8): 1551–1566. doi:10.1038/nprot.2013.092. 10.1038/nprot.2013.092 PMC6519453 23868073

[CR36] Molina J, Sikora M, Garud N, Flowers J, Rubinstein S (2011). Molecular evidence for a single evolutionary origin of domesticated rice. PNAS.

[CR37] Morinaga T, Kuriyama H (1958). Intermediate type of rice in the subcontinent of India and Java. Jpn J Breed.

[CR38] Nei M (1987). Molecular evolutionary genetics.

[CR39] Oka HI (1983) The Indica-japonica differentiation of rice cultivars - a review. In: Proc 4th Int SABRAO Cong, pp 117-128

[CR40] Oka HI (1988). Origin of cultivated rice.

[CR41] Pedregosa F, Weiss R (2011). Brucher M. Scikit-learn: Machine Learning in Python.

[CR42] Peltier M (1970). Les denomination varietales du riz cultivée (Oryza sativa L.) à Madagascar. Journal d’agriculture tropical et de botanique appliquée.

[CR43] Perrier X, Jacquemoud-Collet JP (2006) DARwin software (http://darwin.cirad.fr/darwin)

[CR44] Pritchard JK, Stephens M, Donnelly P (2000). Inference of population structure using multilocus genotype data. Genetics.

[CR45] Radanielina T, Ramanantsoanirina A, Raboin LM, Frouin JF, Perrier X et al (2012) The original feature of rice (*Oryza sativa* L.) genetic diversity and the large effective population size of rice local varieties in the highlands of Madagascar build a strong case for *in situ* conservation. Genet Resources Crop Evol. 10.1007/s10722-012-9837-3

[CR46] Raison JP (1972) Utilisation du sol et organisation de l'espace en Imerina ancienne. Etudes de géographie tropicale offertes à Pierre Gourou. Paris (FR) ; La Haye: Mouton, pp. 407–425

[CR47] Santos J, Chebotarov D, McNally KL, Bartholome J, Droc G (2019). Fine scale genomic signals of admixture and alien introgression among Asian rice landraces. Genome Biol Evol.

[CR48] Schneider S, Roessli D, Excoffier L (2000) Arlequin: a software for population genetics data analysis. Genetics and Biometry Laboratory, Department of Anthropology, University of Geneva

[CR49] Second G (1982). Origin of the genic diversity of cultivated rice (*Oryza* spp.): study of the polymorphism scored at 40 isozyme loci. Jap J Genet.

[CR50] Sharma SD, Tripathy S, Biswal J, Nanda JS (2000). Origin of *O. sativa* and its ecotypes. Rice breeding and genetics: research priorities and challenges.

[CR51] Sweeney M, McCouch S (2007). The complex history of the domestication of rice. Ann Bot.

[CR52] Thomson JM, Septiningsih EM, Suwardjo F, Santoso TJ, Silitonga TS (2007). Genetic diversity analysis of traditional and improved Indonesian rice (*Oryza sativa* L.) germplasm using microsatellite markers. Theor Appl Genet.

[CR53] Tofanelli S, Bertoncini S, Castrı L, Luiselli D, Calafell F (2009). On the origins and admixture of Malagasy: new evidence from high-resolution analyses of paternal and maternal lineages. Mol Biol Evol.

[CR54] Vitte C, Ishii T, Lamy F, Brar D, Panaud O (2004). Genomic paleontology provides evidence for two distinct origins of Asian rice (*Oryza sativa* L.). Mol Gen Genomics.

[CR55] Wang W, Mauleon R, Hu Z, Chebotarov D, Tai S, Wu Z, Li M, Zheng T, Fuentes RR, Zhang F, Mansueto L, Copetti D, Sanciangco M, Palis KC, Xu J, Sun C, Fu B, Zhang H, Gao Y, Zhao X, Shen F, Cui X, Yu H, Li Z, Chen M, Detras J, Zhou Y, Zhang X, Zhao Y, Kudrna D, Wang C, Li R, Jia B, Lu J, He X, Dong Z, Xu J, Li Y, Wang M, Shi J, Li J, Zhang D, Lee S, Hu W, Poliakov A, Dubchak I, Ulat VJ, Borja FN, Mendoza JR, Ali J, Li J, Gao Q, Niu Y, Yue Z, Naredo MEB, Talag J, Wang X, Li J, Fang X, Yin Y, Glaszmann JC, Zhang J, Li J, Hamilton RS, Wing RA, Ruan J, Zhang G, Wei C, Alexandrov N, McNally KL, Li Z, Leung H (2018). Genomic variation in 3,010 diverse accessions of Asian cultivated rice. Nature.

[CR56] Wang Z, Second G, Tanksley S (1992). Polymorphism and phylogenetic relationship among species in the genus *Oryza* as determined by analysis of nuclear RFLPs. Theor Appl Genet.

[CR57] Wright S (1931). Evolution in Mendelian populations. Genetics.

[CR58] Zhao K, Tung CW, Eizenga GC, Wright MH, Ali ML, Price AH, Norton GJ, Islam MR, Reynolds A, Mezey J, McClung AM, Bustamante CD, McCouch SR (2011). Genome-wide association mapping reveals a rich genetic architecture of complex traits in *Oryza sativa*. Nat Commun.

