# Supplementary figures and images for: Evolutionary Processes Involved in the Emergence and Expansion of an Atypical O. sativa Group in Madagascar
Source: Rice (N Y). 2021 May 20;14:44. doi: 10.1186/s12284-021-00479-8 (PMC8137759; doi:10.1186/s12284-021-00479-8)

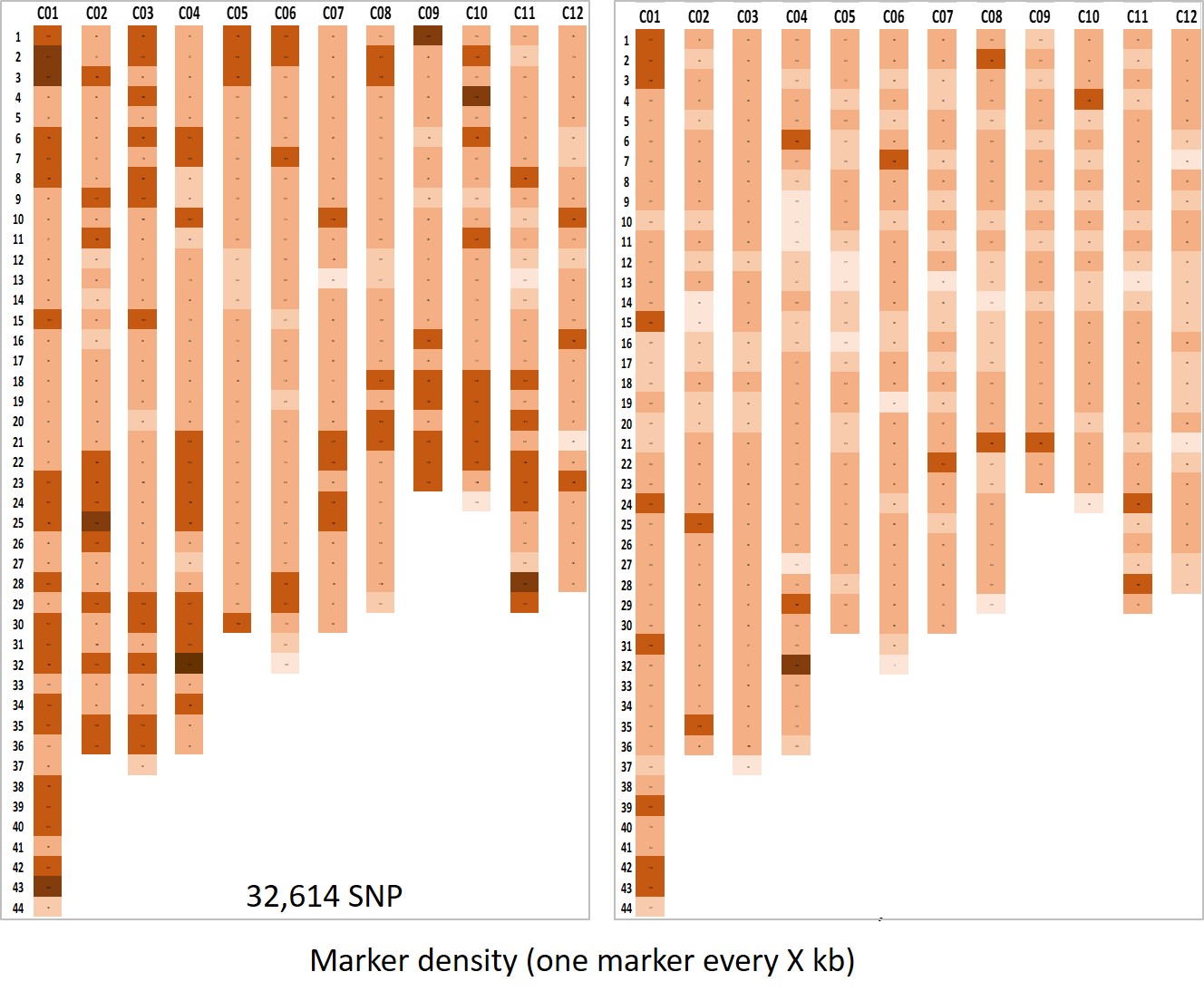

Supplement: Supplementary file 1 — Additional file 1: Supplementary Figure S1: Heat map of distribution of the two set SNP loci (32,614 and 23,981) along the genome. [file 12284_2021_479_MOESM1_ESM.jpg]

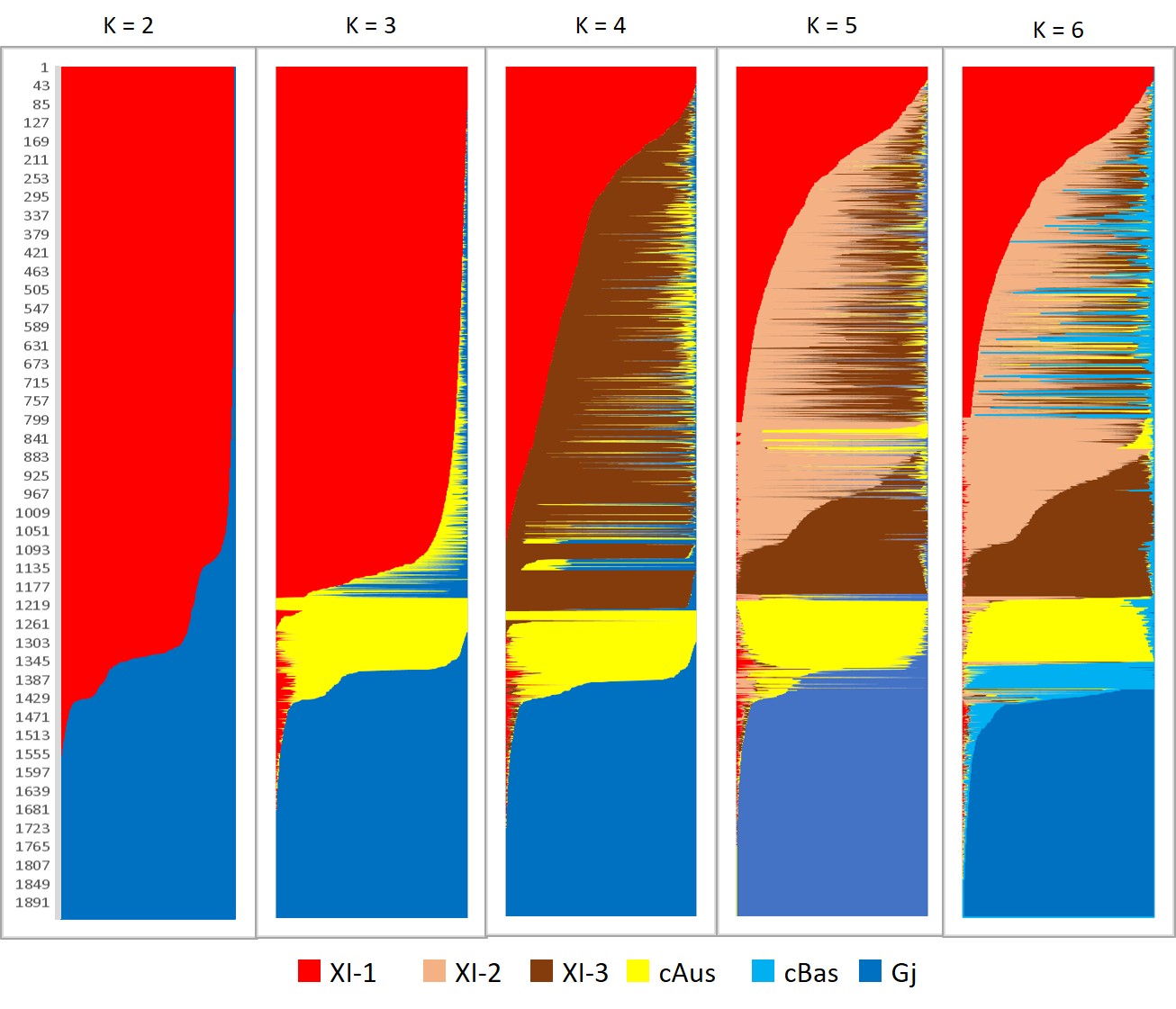

Supplement: Supplementary file 2 — Additional file 2: Supplementary Figure S2: Population structure in 1929 accessions of the Asian panel estimated from 23,981 genome wide SNPs. The subpopulations are coloured according to their membership to groups defined by Wang et al. (2018). [file 12284_2021_479_MOESM2_ESM.jpg]

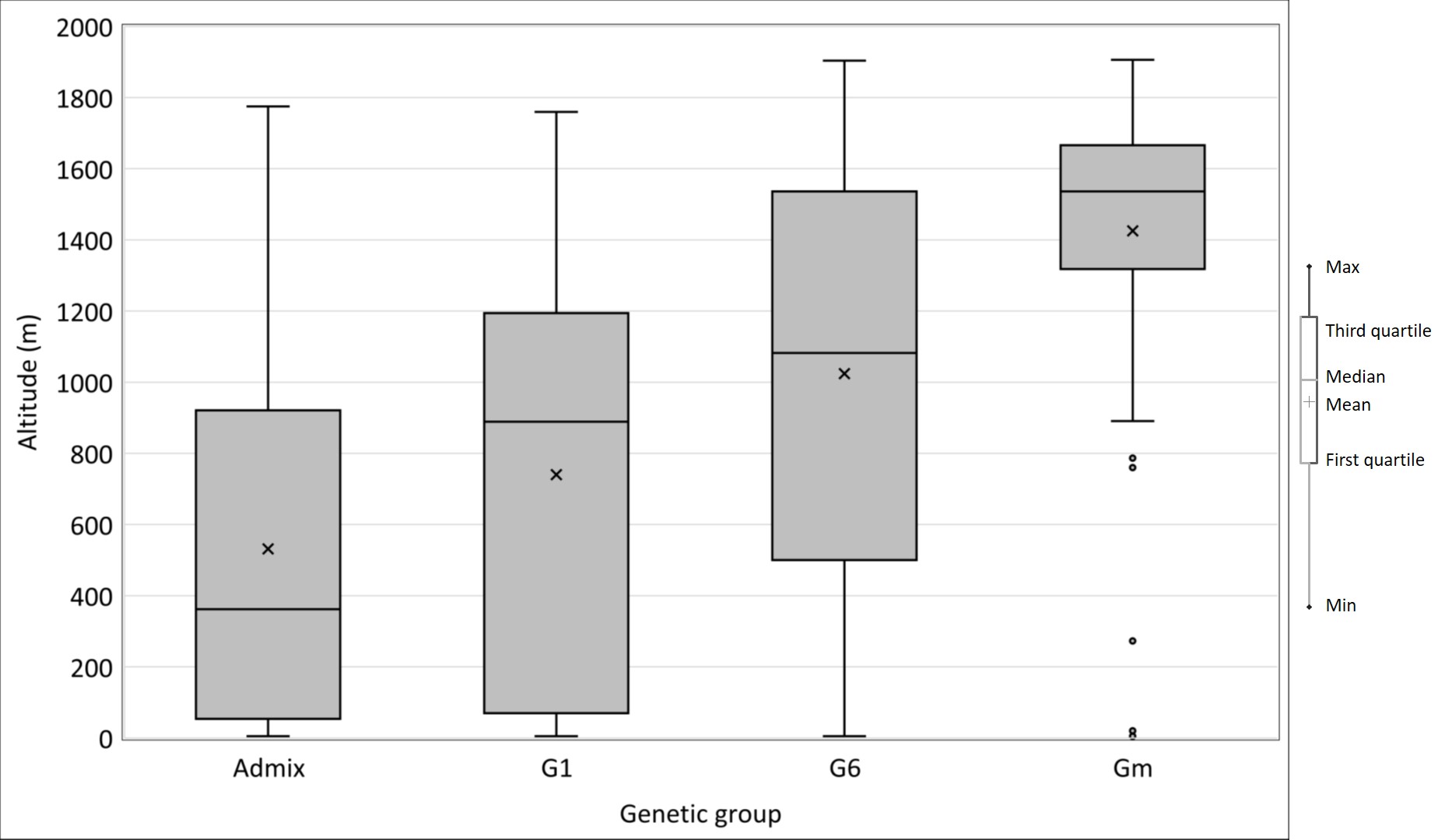

Supplement: Supplementary file 3 — Additional file 3: Supplementary Figure S3: Altitudinal distribution of the Malagasy panel 620 rice accessions according to their membership to groups defined by the sNMF-based analysis of population structure. [file 12284_2021_479_MOESM3_ESM.jpg]

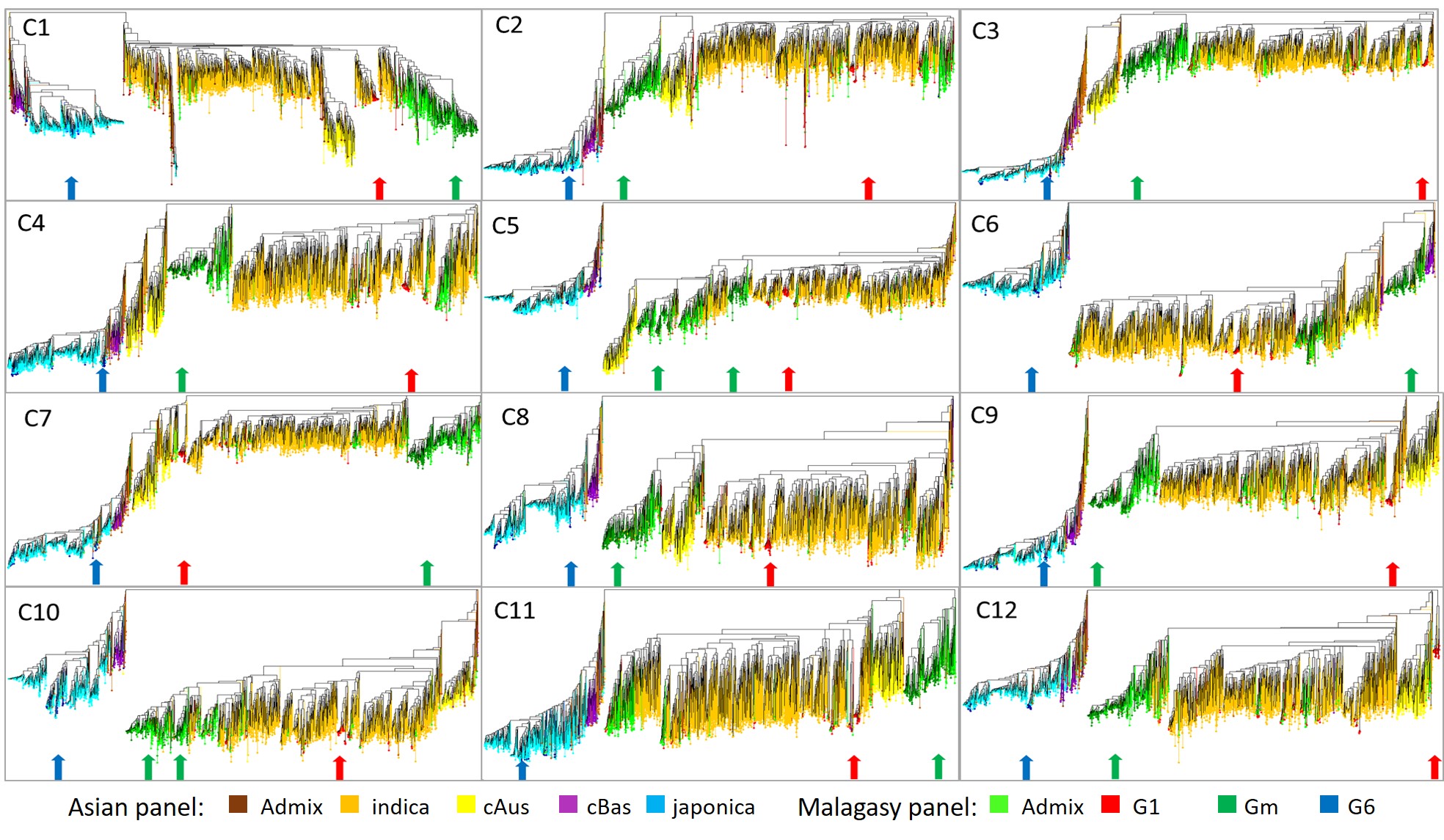

Supplement: Supplementary file 4 — Additional file 4: Supplementary Figure S4: Unweighted neighbour-joining tree of simple matching distances, constructed with the genotypic data of individual chromosomes (C1 to C12). Accessions of the Asian panel are coloured according to their membership to subpopulations defined by Wang et al. (2018). Accessions of the Malagasy panel are coloured according to their membership to sNMF groups at K=3 and ancestry coefficient threshold of 0.8. Positions of the Malagasy accessions on the trees are highlighted with coloured arrows corresponding to their group membership. Accessions of the Asian panel are coloured according to their membership to major populations defined by Wang et al. (2018). [file 12284_2021_479_MOESM4_ESM.jpg]

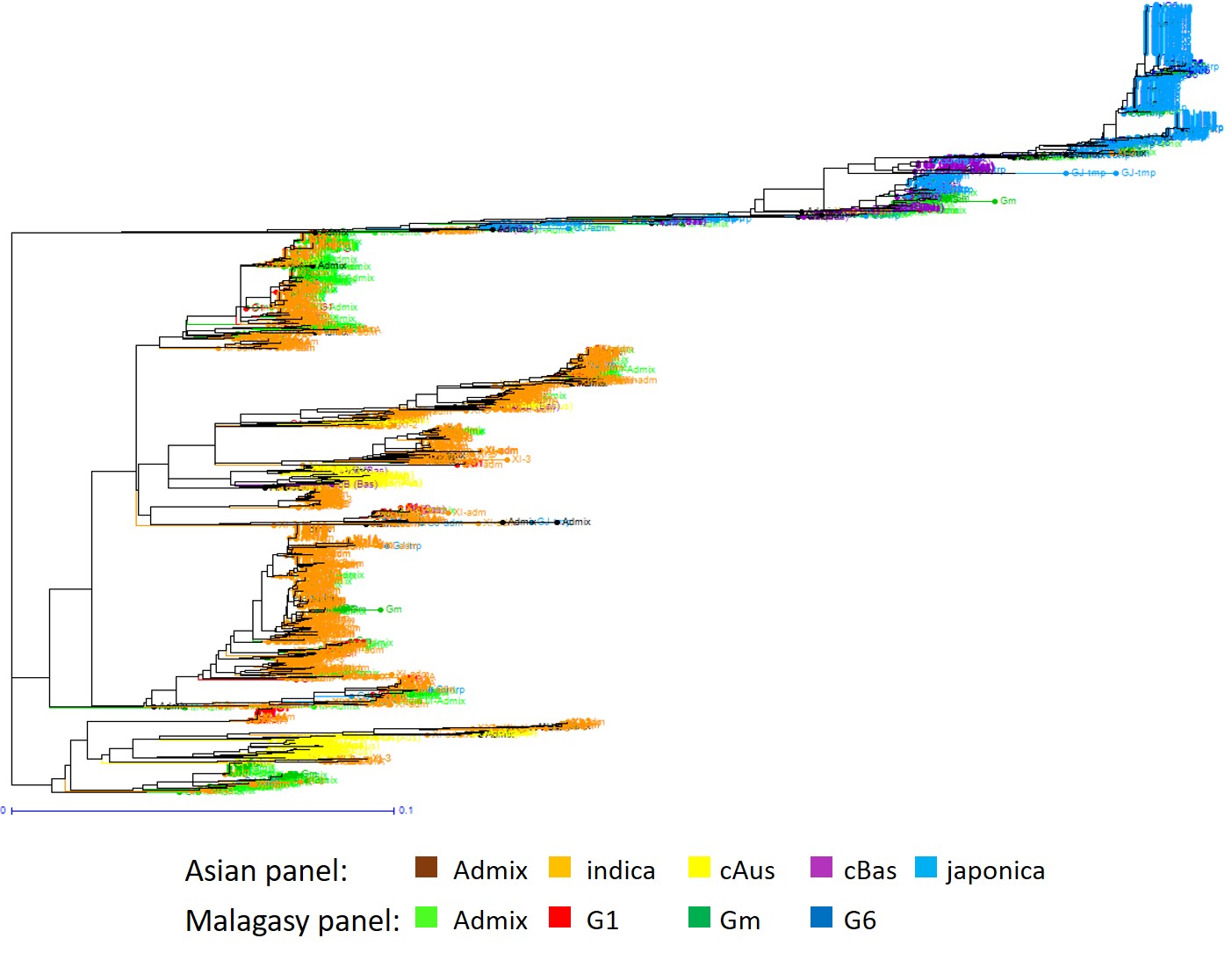

Supplement: Supplementary file 5 — Additional file 5: Supplementary Figure S5: Unweighted neighbour-joining tree of simple matching distances, constructed with the genotypic data at 11.6 – 15.7 Mb segment of chromosome 11 (225 SNP). Accessions of the Asian panel are coloured according to their membership to major populations defined by Wang et al. (2018). Accessions of the Malagasy panel are coloured according to their membership to sNMF groups at K = 3 and ancestry coefficient threshold of 0.8. [file 12284_2021_479_MOESM5_ESM.jpg]

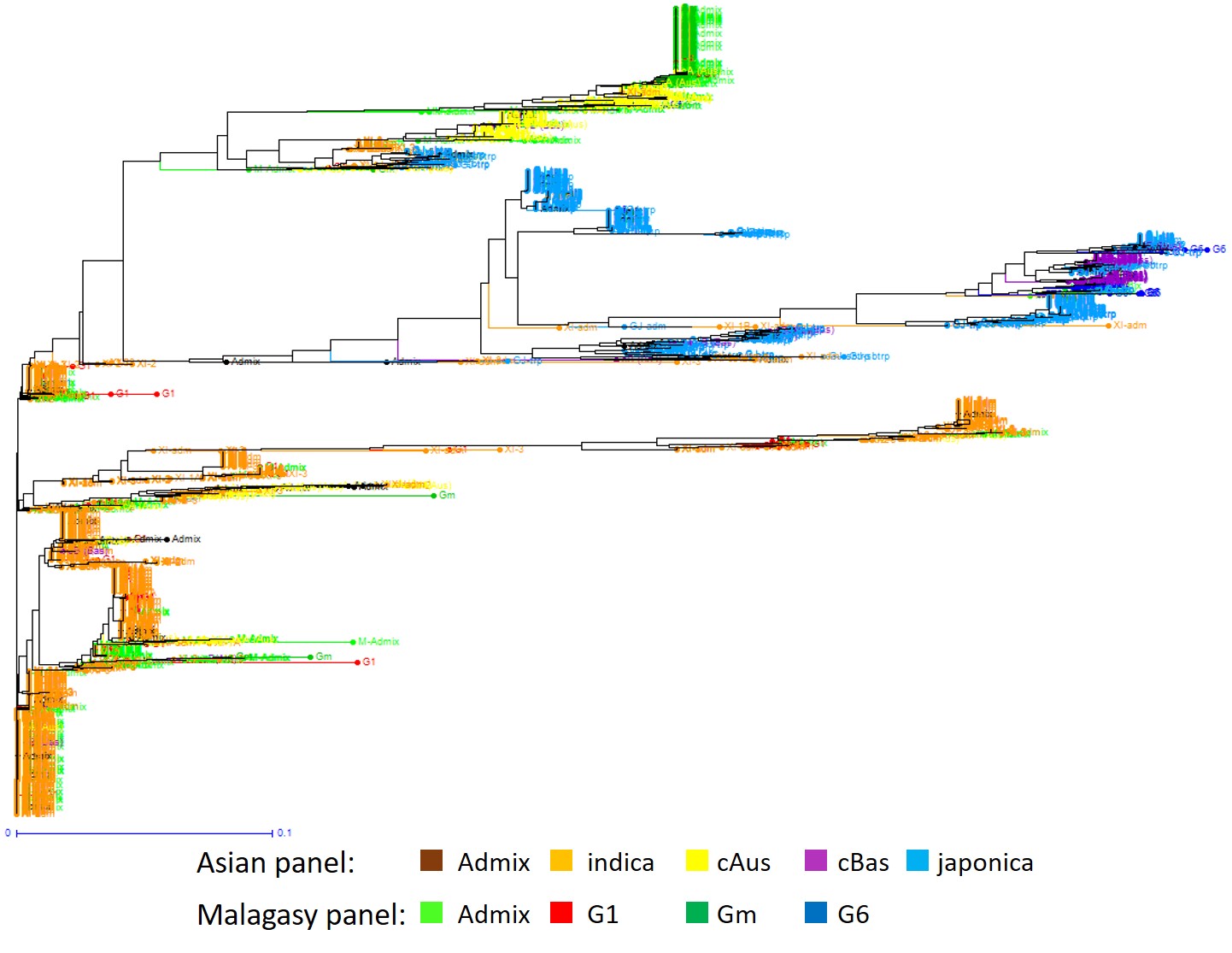

Supplement: Supplementary file 6 — Additional file 6: Supplementary Figure S6: Unweighted neighbour-joining tree of simple matching distances, constructed with the genotypic data at 10.9 – 16.3 Mb segment of chromosome 8 (221 SNP). Accessions of the Asian panel are coloured according to their membership to major populations defined by Wang et al. (2018). Accessions of the Malagasy panel are coloured according to their membership to sNMF groups at K = 3 and ancestry coefficient threshold of 0.8. [file 12284_2021_479_MOESM6_ESM.jpg]
